# Development of a multimodal health education module for dementia prevention in at-risk rural Indian elders: a SMRUTHI INDIA initiative

**DOI:** 10.3389/frdem.2026.1914082

**Published:** 2026-10-01

**Authors:** Kritika Sharma, Aditi Dubey, Shubham Gupta, Kajal Fulara, Hina Narzari, Nilima Nilima, Sneha, Kamini, Sakshi Sharma, Varuna Sharma, Shikha Chaudhary, Phaniraj Vastrad, N. Priya Darshanraj, Sandip Bhattacharjee, Pradyut Datta, Gajraj Singh Shekhawat, Arnav Verma, V. Harshath Ajay, Jajur Rajappa Sandesh, Ambika Y. V., Bishakha Sen, Shalina Jamatia, Priyanka Kumari Meena, Hitesh Tiwari, Shaily Bhushan, Sagar P. M., Ripanjit Singh, Manish Acharjee, Vikram Raigar, Kalpna Sharma, Aishwarya Bhovi, Mailarappa Padiyappa Hooli, Shilpa K., Naveen M.R., Dipankar Deb, Pameli Jamatia, Pooja Sharma, Akhilesh Nagar, Tinku Yogi, Rishav Bhatia, Rishabh Gautam, Maroof Khan, Vaibhav Patil, Rajeev Aggarwal, Ashima Nehra, Kanika Vasudeva, Nimin Hafeez, Chanchal Goyal, Subarna Roy, Manish Barvaliya, Subrata Baidya, Shampa Das, P. K. Anand, Sunil K. Raina, Meenakshi Sharma, R. S. Dhaliwal, Abhik Sinha, Anu Gupta, Venugopalan Y. Vishnu, M. V. Padma Srivastava

**Affiliations:** 1 Department of Neurology, All India Institute of Medical Sciences, New Delhi, India; 2 Department of Biostatistics, All India Institute of Medical Sciences, New Delhi, India; 3 Model Rural Health Research Unit (MRHRU), Raichur, Karnataka, India; 4 Model Rural Health Research Unit (MRHRU), Khumulwng, Tripura, India; 5 Model Rural Health Research Unit (MRHRU), Jaipur, Rajasthan, India; 6 Department of Community Medicine, Dr. RP Government Medical College, Tanda, Himachal Pradesh, India; 7 Department of Psychiatry, All India Institute of Medical Sciences, New Delhi, India; 8 Neuro-Physiotherapy Unit, All India Institute of Medical Sciences, New Delhi, India; 9 Department of Neuropsychology, All India Institute of Medical Sciences, New Delhi, India; 10 Indian Council of Medical Research (ICMR) Headquarters, New Delhi, India; 11 Department of Health Research, Government of India, New Delhi, India; 12 Indian Council of Medical Research National Institute of Traditional Medicine (ICMR-NITM), Belagavi, Karnataka, India; 13 Agartala Government Medical College, Agartala, Tripura, India; 14 Indian Council of Medical Research-National Institute of Health Research, Jodhpur, Rajasthan, India

**Keywords:** at-risk elderly, dementia prevention, lifestyle intervention, primary care intervention, rural India

## Abstract

**Introduction:**

SMRUTHI-India (Strategic Multimodal Intervention in at-risk Elderly Indians for Prevention of Dementia), was initiated across rural India to establish a cohort of at-risk elderly for primary dementia prevention. Dementia prevalence being 7.4% and higher rates in rural areas, culturally tailored psychoeducational materials are essential. The current study thus aimed to construct and validate a multimodal intervention in elderly.

**Methods:**

A multimodal intervention “Care Bundle Module (CBM)” booklet was developed, followed by content validation. It was pilot tested for feasibility and preliminary intervention effects in hospital setting (central site) using cohort multiple randomised controlled trial (cmRCT) design. Using purposive sampling, some high-risk participants were also selected from periphery sites and intervention was conducted using pre-post design to assess feasibility, usability, and acceptability in community setting. Change in Lifestyle for Brain health (LIBRA) index was assessed. Simultaneously, a booklet containing ten themes was developed into thematic animated videos, and revised based on behavioural science expert feedback. CBM was assessed for understandability and actionability using Patient Education Material Assessment ToolPrint/Audio-Visual (PEMAT-P/AV).

**Results:**

Intervention was developed in three languages (Hindi, Kannada, Bengali). In hospital setting and across periphery sites respectively, median (IQR) age was 61 (57, 66) years, mean age: 64.9 ± 5.8 years; median LIBRA: −4.6 (*p* = 0.02) and −1.0 (*p* = 0.001) (intervention arms). Rural setting showed higher recruitment and retention rates with good adherence. Between 70 to 96%, participants rated booklet and videos as well understandable and actionable on PEMAT.

**Conclusion:**

CBM demonstrated high content validity, acceptability, and adherence across rural settings. This suggests feasibility to conduct multidomain interventions for at-risk elderly to foster healthy ageing.

**Clinical trial registration:**

https://ctri.nic.in/Clinicaltrials/pmaindet2.php?EncHid=ODYxODY=&Enc=&userName=, Identifier CTRI/2024/01/061172.

## Introduction

Dementia is a chronic neurodegenerative disease with significant health and socioeconomic concerns. India has approximately 104 million older adults, with 7.4% prevalence of dementia affecting nearly 8.8 million of Indian geriatric population. The prevalence of modifiable risk factors of dementia is relatively high in elderly (Lee et al., 2023). Considering huge morbidity and mortality rates, lack of widespread availability of disease-modifying drugs, and long-term care infrastructure in LMICs, there is a need for primary interventions to delay/prevent the onset of dementia.

The FINGER Study was the first long-term study to investigate efficacy of multidomain lifestyle interventions in older at-risk populations, to prevent cognitive decline (Kivipelto et al., 2013). After two-year intervention, intervention group showed greater improvement than control group on composite measure of cognition. Similarly, U.S. POINTER adapted an intervention based on the FINGER study, which was culturally relevant (Baker et al., 2024). Likewise, other trials have been conducted (Appendix: p. 2-10) in Western countries, where effectiveness of intervention may be underestimated due to high standards of usual care. Recent studies like SANSCOG examined the impact of protective and risk factors on dementia onset (Ravindranath and SANSCOG Study Team, 2023). But there are limited studies targeting primary prevention of dementia in India. We need pragmatic and culturally appropriate multidomain interventions targeting primary care by reducing modifiable risk and increasing protective factors as per needs and resources of our population.

The “Strategic Multimodal Intervention in at-risk Elderly Indians for Prevention of Dementia: SMRUTHI INDIA” is an ongoing cohort study of rural population across four Indian zones (Himachal Pradesh-North, Rajasthan-West, Tripura-East, Karnataka-South) (Gupta et al., 2025). Based on the established cohort, aim of this study was to develop and validate a multimodal intervention for primary prevention of dementia in the form of a booklet and videos (Care Bundle Module-CBM), evaluate feasibility and acceptability using qualitative data and quantitative tools [Patient Educational Material Assessment Tool (PEMAT)] (Shoemaker et al., 2014). The study also aimed to assess the feasibility, usability, and acceptability of intervention in a cmRCT design at the central site (included a control arm) and pre-post intervention design in the peripheral or community site (no control arm).

Some studies suggest that print material with audio-visual content may reinforce adaptive learning, particularly among older adults, thereby facilitating behaviour change (Paragas and Barcelo, 2019; Park et al., 2020; Wischer et al., 2018).

## Methods

The present study involved the development of CBM booklet, followed by content validity and assessment of understandability and actionability (PEMAT-Print). It was tested in hospital and rural settings (Figure 1). To assess the feasibility of the intervention, the pilot study conducted at the central site employed a methodology modelled on the original SMRUTHI cohort. This methodology follows a cohort multiple randomised controlled trial (cmRCT) design, in which participants are randomly assigned to the intervention arm. Some high-risk elderly participants [Cardiovascular Risk Factors, Ageing and Incidence of Dementia (CAIDE) score ≥ 6] were selected at periphery sites using purposive sampling.

**Figure 1:**
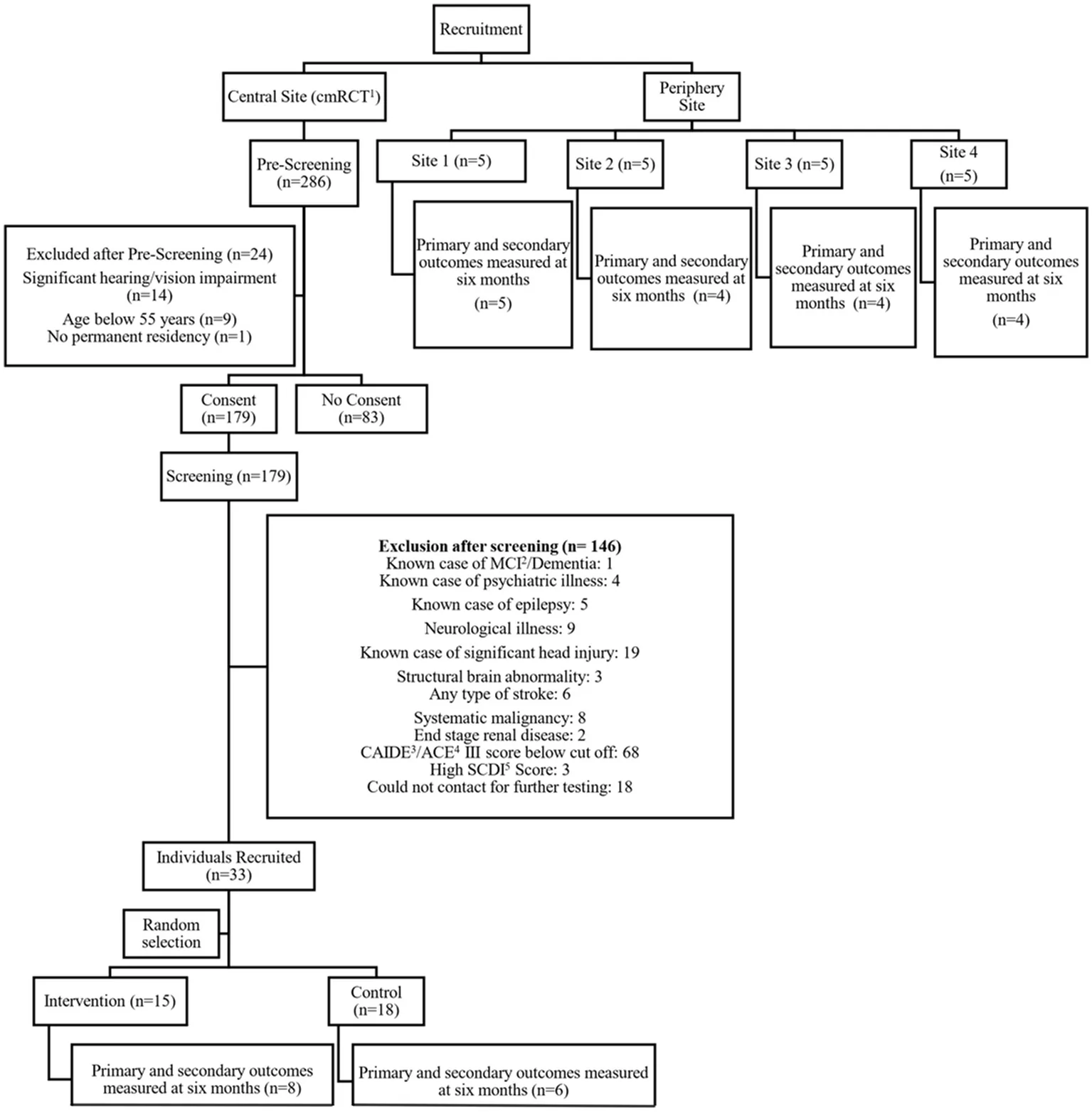
SMRUTHI pilot consort diagram. cmRCT, Cohort Multiple Randomised Control Trial; MCI, Mild Cognitive Impairment; CAIDE, Cardiovascular Risk Factors, Ageing, and Incidence of Dementia; ACE III, Addenbrooke’s Cognitive Examination; SCI, Subjective Cognitive Decline.

The booklet was converted into thematic animation videos catering to rural population. The videos were shared with a behavioural science expert, and feedback was incorporated. Then, PEMAT- Audio-visual (AV) was administered to some participants from cohort to assess actionability and understandability.

CBM development is explained in subsequent sections.

### Theoretical framework of intervention

The Health Behaviour Model (HBM) is a theory of health behaviour that focuses on people’s attitudes and beliefs to explain health behaviours (Glanz et al., 1990). This study focused on increasing awareness of dementia, perceived severity of symptoms in daily life, perceived advantages of taking actions to manage risk and enhance protective factors, and strengthening belief to adapt healthy lifestyle changes, which could result in enhanced efforts towards risk reduction and preventing cognitive decline (Appendix: p. 26).

### Care bundle module development (booklet)

The preliminary phase included developing CBM booklet, prepared by a team comprising neurologists, neuropsychiatrist, neuropsychologist, psychologists, dietitian, and physiotherapists from the central site.

#### Preparatory stage

An extensive review was conducted on databases (PubMed, JSTOR, EMBASE) and four major themes for content curation were identified (Appendix: p. 22) and first draft of the booklet was created (English). A Supplementary Video summarising the purpose and content of booklet was prepared to ensure uniform delivery of instructions to the participants of pilot feasibility study.

#### Acceptability and adaptability stage

The first draft of the booklet was presented to stakeholders (periphery sites), and feedback was incorporated. Further, the booklet was sent to ten experts, along with a questionnaire (Appendix: p. 13–14), of which five responded. Based on experts’ feedback (Appendix: p. 27), the booklet was revised. The final draft of CBM was translated to three languages (Hindi, Bengali and Kannada).

#### Content validity and PEMAT

The booklet was sent to the same experts after incorporating their feedback for further evaluation. The booklet was revised accordingly and content validity indices (CVI) were calculated. PEMAT-P was used to assess booklet’s actionability and understandability on 83 participants from ongoing cohort (different from pilot participants) after obtaining written informed consent. Each item was rated by participants on a ternary scale of either “Yes,” “No” or “Not-Applicable (N/A).” Also, qualitative feedback (Appendix: p. 16) was taken from the participants through a focused-group discussion.

### Pilot feasibility testing using CBM booklet

#### Study participants and recruitment

Enrolment for pilot cmRCT commenced on April 12, 2024. The study sample for periphery sites was derived from SMRUTHI cohort, whose recruitment was started on July 27, 2023. The recruitment for study sample at central site was started on November 24, 2023. In this study, designed to evaluate the feasibility, usability, and acceptability of the intervention, a formal sample size calculation was not performed. The sample size was determined in accordance with the recommendations for pilot studies (Julious, 2005; Relton et al., 2010). Literature suggests a minimum of 12 participants per group as a rule of thumb for pilot studies to obtain preliminary estimates of study parameters. Accordingly, we recruited a total of 50 participants to allow assessment across both central and peripheral study settings while accounting for potential loss to follow up. The participants at central site included individuals aged ≥55 years, CAIDE score ≥6, and normal cognition (no complaints on Subjective Cognitive Decline – Interview and normal performance on Addenbrooke’s Cognitive Examination III/ACE III) at baseline (Figure 1). Individuals diagnosed with dementia/mild cognitive impairment (MCI), significant vision/hearing impairment, communication problems, head injury (deemed to impact neuropsychological testing), any type of stroke, known psychiatric illness (including psychosis/intellectual disability/major depressive disorder), structural brain abnormality, significant medical illness (decompensated liver disease, end stage renal disease, systemic malignancy), and epilepsy were excluded. The periphery sites selected literate participants fulfilling same criteria. A detailed neuropsychological assessment was conducted on the recruited participants. These assessments included ACE III, Indian Council of Medical Research-Neurocognitive Tool Box/ICMR-NCTB [Verbal Learning Test (VLT), Test des Neuf Images – 93 (TNI-93), Trail Making Test (TMT), Category Fluency Test (CFT), Picture Naming Test (PNT), and Stick Design Test (SDT)]. Details of these assessments are shared in previously published papers (Gupta et al., 2025; Nilima et al., 2026). Additional consent was obtained from participants in the intervention group.

#### Intervention arm

The intervention arm was provided with the booklet and orientation was done by a team of field investigators and psychologists using an explanatory video. The outcome assessors were blinded to the participants receiving intervention/care as usual. Monthly follow-ups were conducted for a period of 6 months. Adherence was measured at third and sixth months using Post-Intervention Adherence Scale (PIAS) (Appendix: p. 19–21). At 6 months, participants underwent a follow-up neuropsychological assessment. Any unintended harm during performing or consuming suggested physical activities/diet were asked to be reported for the team to monitor.

#### Control arm

The control arm received routine health advice in the form of a health awareness video. These participants were also followed for 6 months, along with monthly follow-ups and at the end of 6 months, neuropsychological assessment was conducted.

#### Outcomes

The primary outcome included change in the Lifestyle for Brain Health Index/LIBRA (population based predictive instrument that assesses cognitive risk factors in general population) at 6 months, and secondary outcomes included preliminary effects and feasibility of intervention (Deckers et al., 2015; Vos et al., 2017). Feasibility included recruitment rate (pre-screening criteria: age >55 years, no significant hearing/vision impairment, permanent Indian resident), enrolment rate (who provided informed consent, were eligible after completing screening; ≥80%), retention rates (completing six-month trial; <20% dropout), and adherence to intervention [percentage following at least two of five self-reported components: aerobic activity (5 days/week), cognitive activities (two/week), vascular risk monitoring, dietary improvement, addiction cessation]. Intervention effect was defined as ≥50% participants achieving at least two of six targets:

Mediterranean-DASH Intervention for Neurodegenerative Delay (MIND Diet) score ≥8, Global Physical Activity Questionnaire (GPAQ) > 600 Metabolic Equivalent Task (METS), Fagerström Test for Nicotine Dependence (FTND) < 5, Cut down, Annoyed, Guilty, and Eye-opener- Adapted to include Drugs (CAGE-AID) < 1, blood pressure (BP) < 140/90 mmHg, or HbA1c reduction of 1% over 6 months.

### Care bundle module development (videos)

The CBM Videos were developed and validated, incorporating the content of CBM Booklet to facilitate easier delivery, specifically to illiterate population.

#### Pre-production stage

The content of the Booklet (Psychoeducation, Modifiable Risk Factors, Protective Factors and Additional Information) was converted into ten textual scripts (Hindi language) following a relatable storyline of an older adult in an Indian rural setting developing early signs of dementia. The scripts were also translated and culturally modified into two other Indian languages (Kannada and Bengali). These were developed into storyboards elaborating on characters, on-screen text/images, and background sounds.

#### Production stage

The second stage involved synthesis and implementation of scripts and storyboards into animated videos. In this stage, voice-overs of animated characters were recorded, illustrative images, colours, and sounds were chosen.

#### Post-production stage

The final stage included edits, feedback from target population and validation of the video content. To collect feedback, the videos were shown to participants across periphery sites and qualitative feedback was collected on their culture, experiences, and everyday life. PEMAT-AV was used to assess understandability and actionability of the videos on 86 participants (different from pilot participants) along with qualitative feedback (Appendix: p. 17-18). Further, feedback was taken from a behavioural science expert who suggested adding caricatures, incorporating theory of behavioural change, modifying some medical jargon, and adding key messages at the end of each video and was incorporated.

### Statistical analysis

CVI was calculated using expert feedback on CBM booklet at both item (Items shared with experts) and scale levels. Using Simple Measure of Gobbledygook (SMOG) index, readability grade level of booklet was determined (Grabeel et al., 2018; Mc Laughlin, 1969). Understandability and actionability were evaluated using PEMAT. In pilot feasibility study, to assess differences between pre- and post-intervention scores, change in measures were investigated using paired samples *t*-test (and summarised using mean ± SD) for normally distributed measures including MIND diet score, QOL, ACE III, and other ICMR-NCTB measures viz. VLT (TL, Delayed Recognition), TNI-93 (Total & Spatial Recall), CF, PNT, score (for copy, immediate, and delayed) recall, and SDT score. Pre- post assessment for other measures was analysed using Wilcoxon’s signed rank test [and summarised using median (Interquartile range/IQR: percentile 25, percentile 75)].

Measures at peripheral site were summarised and analysed in similar manner as that for the central site. The study protocol and statistical analysis plan were published and are freely available (Gupta et al., 2025). Moreover, analyses were performed using complete-case data; participants who did not complete the follow-up assessment were excluded from the outcome analyses. No imputation of missing values was undertaken.

### Funding source

The study progress is monitored by task force of external experts created by Indian Council of Medical Research.

## Results

### Development of CBM

The CBM booklet comprises 127 pages, including psychoeducation of preclinical phase, MCI, dementia, and modifiable factors associated with dementia. The initial draft of booklet had an average Write Item-level CVI of 0.933, I-CVIs ranging from 0.6 to 1.0. Sixteen of the twenty-one items scored 1.0, while five scored lower. Based on expert feedback, two items with I-CVI < 0.7 and three items with I-CVI 0.7–0.99 were revised. The revised version achieved a Scale-level CVI/Average of 0.977. Fleiss’s multi-rater kappa indicated “fair” agreement for two items initially, improving to “excellent” for 19 items after revisions (Appendix: p. 23–24, p. 27). The SMOG index of booklet was 10.07. Understandability and actionability of the booklet was rated variably by the participants: 83 (30.1%) rated understandability between 69% to 94.1% as rated by 30.1% (*n* = 83) participants while 83 (12.1%) rated actionability between 71.4% to 85.7%. The rest gave a rating of 100%.

### Pilot feasibility study

#### Baseline sample characteristics

As shown in Table 1, a total of 33 participants were enrolled at central site and 20 at periphery sites. Participants from central site had a median age of 61 years (IQR 57, 66) and higher median education [15 years (9, 17)] compared to periphery sites, whose mean age was 64.9 ± 5.8 years and median education was 9.5 years (7, 10.8). Participants at central site had a median Body Mass Index (BMI) of 24.2 (21.6, 28.7), Systolic Blood Pressure (SBP) 144 (130, 164), and Diastolic Blood Pressure (DBP) 85 (77, 91). The participants of periphery sites also showed higher mean BMI (25.4 ± 4.7 kg/m^2^), SBP (148.4 ± 25.6 mm Hg), DBP (89.4 ± 10.5 mm Hg), and median LIBRA index (sites: 0.55 [−0.7, 1.6]; central site: −2.6 [−3.8, 0.2]). CAIDE scores were similar between groups (central site: 7.5 ± 1.4; periphery sites: 7 [6.8, 9]).

**Table 1:** Mean ± SD/median [IQR] for sociodemographic characteristics of participants.

Demographic	Central site			Periphery sites (*n* = 20)
	Intervention (*n* = 15)	Control (*n* = 18)	Total (*n* = 33)	
Age (in years)	59.5 (56.2, 63.7)	61.5 (58, 71.7)	61 (57, 66)	64.8 ± 5.8
Education (in years)	15 (11.2, 16.5)	15 (6.2, 17)	15 (9, 17)	9.5 (7, 10.7)
CAIDE score	7.21 ± 1.3	7.7 ± 1.5	7.5 ± 1.4	7 (6.7, 9)
BMI (kg/m^2^)	23.8 (20.9, 29.1)	24.4 (22.8, 27.5)	24.2 (21.6, 28.7)	25.3 ± 4.7
SBP (mm Hg)	130 (118, 157.5)	150.5 (133.5, 164.2)	144 (130, 164)	148.3 ± 25.6
DBP (mm Hg)	81 (77, 96.5)	86 (77.5, 90.7)	85 (77, 91)	89.3 ± 10.5
Libra Index	−3.0 (−4.6, 0.2)	−0.5 (−3.7, 1.1)	−2.6 (−3.8, 0.2)	0.5 (−0.7, 1.6)

#### Primary outcome

At central site, in intervention group (*n* = 8), LIBRA index improved significantly from −3.0 (−4.6, 0.2) to −4.6 (−5.4, −4.55; *p* = 0.02) (Table 2). The control group (*n* = 6) demonstrated an improvement from −0.55 (−3.8, 1.5) to −5.9 (−5.9, −4.3), but was not statistically significant (*p* = 0.19). The median LIBRA index (*n* = 17) improved from 0.55 (−0.8, 1.7) to −1.0 (−1.4, 0.6; *p* = 0.001) at periphery sites (Table 3). These findings suggest that intervention contributed to meaningful reductions in lifestyle-related risks in intervention arms.

**Table 2:** Mean ± SD/median scores of lifestyle factors and neurocognitive assessments at baseline and follow-up in the intervention and control group (central site).

Pilot test	Intervention group				Control group			
	Baseline (*n* = 15)	6 month follow up (*n* = 8)	*p*-value	∆ (95% CI)	Baseline (*n* = 18)	6 month follow up (*n* = 6)	*p*-value	∆ (95% CI)
Primary outcome								
LIBRA score	−3.0 (−4.6, 0.2)	−4.6 (−5.4, −4.5)	0.02	1.55 (0.1, 5.9)	−0.55 (−3.8, 1.5)	−5.9 (−5.9, −4.3)	0.19	1.6 (−1.4, 4.7)
Secondary outcomes								
GPAQ total activity (minutes/week)	290 (30, 510)	420 (338, 578)	0.30	−92.5 (−407.6, 255.4)	345 (140, 525)	210 (160, 350)	0.10	142.5 (−68.5, 526)
GPAQ total activity METS	885 (30, 2,445)	1,522.5 (1,220, 2,222.5)	0.40	−180 (−2,478.7, 1723.5)	595 (360, 1,680)	840 (590, 980)	0.29	780 (−464.5, 5,519.2)
GPAQ time spent in sedentary activity (minutes/day)	945 (880, 975)	907.5 (900, 940)	0.50	−2.5 (−198.2, 22.1)	970 (880, 1,000)	980 (930, 1,050)	0.60	−47.5 (−298.6, 228)
MIND diet score	10.2 ± 2.2	10.5 ± 2	0.30	−0.75 (−2.4, 0.9)	9.2 ± 1.9	10.7 ± 2	0.06	−1.34 (−2.8, 0.1)
QoL SF-36 score	705 (640.5, 748)	739.7 (686.7, 754)	0.10	−18.5 (−76.5, 10.6)	702.3 (678, 712)	712.5 (629.5, 761)	0.40	40.5 (−76.2, 96.1)
QoL physical functioning	93.3 ± 7.5	100 ± 0	0.03	−5.6 (−10.3, −0.9)	92.5 ± 9.3	99.1 ± 2	0.10	−3.3 (−7.6, 0.9)
Role functioning/Physical (*n* = 14)	95 ± 10.4	100 ± 0	0.20	−6.25 (−15.9, 3.4)	98.6 ± 5.9	100 ± 0	-	0 (0, 0)
Role functioning/Emotional (*n* = 14)	97.8 ± 8.6	75 ± 11.3	<0.001	25 (15.5, 34.4)	94.2 ± 17.9	68.3 ± 6	0.03	31.6 (25.3, 38)
Energy/Fatigue (*n* = 14)	72.3 ± 12.2	75 ± 11.3	0.40	−4.4 (−15.6, 6.9)	70.8 ± 11.4	68.3 ± 6	0.60	1.7 (−5.5, 8.8)
Emotional well-being (*n* = 14)	79.5 ± 14.1	86.6 ± 3.7	0.20	−6.1 (−17.5, 5.3)	78.4 ± 19.9	81.3 ± 17.1	0.40	3.3 (−24.6, 31.2)
Quality of Life (QoL) social functioning	90.8 ± 18	93.7 ± 13.4	0.40	4.7 (−7.7, 17)	97.2 ± 9.2	98 ± 5.1	0.30	2.1 (−3.2, 7.4)
QoL pain	81.8 ± 23.7	82.8 ± 19.2	0.50	5 (−11.6, 21.6)	84.3 ± 12.5	83 ± 19.6	0.80	2.1 (−18.7, 22.8)
QoL general health	78.3 ± 19.1	80 ± 14.9	0.99	0 (−11.8, 11.8)	78.3 ± 15.7	80 ± 6	0.10	7.5 (−3.4, 18.4)
ACE III	92.6 ± 4	95.3 ± 2.1	0.20	−1.6 (−4.7, 1.4)	90.6 ± 6.7	93.1 ± 2.5	0.10	−1.8 (−4.6, 0.9)
ICMR NCTB VLT total learning (TL) (T1 + T2 + T3)	17.7 ± 3.4	19.8 ± 4.2	0.07	−1.6 (−3.4, 0.2)	15.2 ± 5.3	18.7 ± 4.1	0.40	−1.7 (−6.3, 3)
ICMR NCTB VLT LOT	4 (3, 7)	5 (4, 6)	0.20	−1 (−3.9, 1.3)	5 (3, 6)	4 (1, 6)	0.50	0.5 (−5.5, 10.5)
ICMR NCTB VLT delayed recall	4 (3, 6)	5 (5, 7)	0.06	−1.5 (−2.3, 0.3)	5 (1, 6)	7 (6, 7)	0.60	−1 (−2.9, 2)
ICMR NCTB VLT delayed recognition	18.3 ± 1.6	18.8 ± 0.7	0.70	−0.25 (−1.5, 1.1)	17.7 ± 2	19.3 ± 1.2	0.50	−0.5 (−2.2, 1.2)
ICMR NCTB TNI-93 total recall (Free recall + Cued recall)	8.9 ± 0.5	8.8 ± 0.7	0.99	0 (−0.9, 0.9)	8.8 ± 0.7	9 ± 0	-	0 (0, 0)
ICMR NCTB TNI-93 spatial recall	7.8 ± 1.8	8 ± 1	0.30	0.4 (−0.4, 1.1)	6.7 ± 2.4	8.7 ± 0.8	0.20	−2 (−5.2, 1.2)
ICMR NCTB trail making test part A	53 (41, 64)	57 (42, 76)	0.10	−19 (−33.8, 11.2)	82 (51, 105)	84 (58, 105)	0.60	−7 (−61.3, 45.9)
ICMR NCTB trail making test part B	160 (121, 185)	128 (110, 152)	0.20	9 (−7.9, 28.3)	188 (149, 300)	228 (162, 244)	0.30	20 (−26.3, 117.3)
ICMR NCTB category fluency (CF) vegetables	14.7 ± 3.8	15.9 ± 3.7	0.99	0 (−3.1, 3)	13.6 ± 3	14.2 ± 5	0.60	1 (−3.5, 5.5)
ICMR NCTB CF animals	13.8 ± 2.4	13.8 ± 3	0.90	−0.3 (−3.4, 3)	11.8 ± 2.3	11.2 ± 1.6	0.70	0.2 (−1.1, 1.4)
ICMR NCTB CF food items	13.5 ± 3.4	12.8 ± 4.6	0.50	1.6 (−3.2, 6.5)	11.7 ± 3.5	10.7 ± 2.5	0.20	2.2 (0.4, 4)
ICMR NCTB picture naming test	85.6 ± 3.7	85.5 ± 4.8	0.20	1.7 (−1.4, 4.9)	82.6 ± 10	87.2 ± 4.3	0.60	−2.2 (−4.5, 0.2)
Time taken to copy in stick design test (in seconds)	236 (185, 287)	192 (161, 256)	0.90	16 (−79.1, 92.2)	189 (158, 236)	158 (130, 175)	0.20	36.5 (−50.8, 81.9)
Score for Copy	9 ± 1.6	9.6 ± 0.7	0.60	0.1 (−0.4, 0.7)	8.8 ± 2.2	9.8 ± 0.4	0.40	−0.8 (−3, 1.3)
Time taken for immediate recall in stick design test (in seconds)	186 (145, 233)	191.5 (132, 262)	0.20	−26 (−110.1, 38.3)	167.5 (147, 206)	148 (113, 192)	0.50	−9.5 (−81, 68.6)
Score for immediate recall	8.6 ± 1.9	9.4 ± 0.7	0.99	0 (−0.4, 0.4)	8.4 ± 2.3	9.5 ± 0.8	0.50	−0.7 (−3, 1.7)
Time taken for delayed recall in stick design test (in seconds)	160 (134, 227)	171.5 (127.5, 252.5)	0.40	−6.5 (−66.5, 31.2)	176 (134, 228)	160 (112, 214)	0.80	5 (−24.3, 105.9)
Score for delayed recall	9 ± 1.5	9.5 ± 0.8	0.80	0.1 (−0.8, 1.1)	8.7 ± 2.1	9 ± 0.9	0.70	0.3 (−1.5, 2.2)
Stick design score – Total	27 ± 4.7	28.5 ± 1.9	0.60	0.4 (−1.2, 1)	26 ± 6.4	28.3 ± 1.9	0.60	−1.2 (−7.4, 5)

**Table 3:** Mean ± SD/median scores of lifestyle factors and neurocognitive assessments at baseline and follow-up with effect sizes at follow-up (periphery sites).

Pilot test	Baseline (*n* = 20)	6 month follow up (*n* = 17)	*p*-value	∆ (95% CI)
Primary outcome				
LIBRA score	0.5 (−0.8, 1.7)	−1 (−1.4, 0.6)	0.001	−1.1 (−1.8, 0)
Secondary outcomes				
GPAQ total activity (minutes/week)	555 (245, 1,095)	690 (280, 1,050)	0.30	0 (0, 120)
GPAQ total activity METS	1,690 (315, 4,935)	1740 (480, 3,680)	0.90	0 (−386.0, 118.9)
GPAQ time spent in sedentary activity (minutes/day)	932.5 (855, 1,000)	875 (800, 975)	0.06	−15 (−75, 0)
MIND diet score	9.3 ± 1.2	9.6 ± 1.4	0.10	−0.4 (−1, 0.1)
QoL SF-36 score	689.5 (635.5, 736.5)	670 (572, 728)	0.30	0 (−83.7, 10.7)
QoL physical functioning	82.2 ± 15.3	76.8 ± 31.2	0.50	4.7 (−11.2, 20.6)
Role functioning/physical (*n* = 14)	95 ± 17.4	92.6 ± 24.6	0.70	0 (0, 0)
Role functioning/emotional (*n* = 14)	100 ± 0	95.9 ± 10.2	0.10	4.1 (−1.1, 9.3)
Energy/Fatigue (*n* = 14)	74.5 ± 15.2	73.5 ± 19.1	0.80	0.9 (−7.2, 8.9)
Emotional well-being (*n* = 14)	82.5 ± 13.3	86.4 ± 3.5	0.10	−4.8 (−12.1, 2.5)
QoL social functioning	88.2 ± 20.5	88.5 ± 20	0.50	−2.3 (−9.8, 5.1)
QoL pain	82 ± 22.6	79.1 ± 17.2	0.90	−0.3 (−11.6, 10.9)
QoL general health	68.2 ± 16.1	69.7 ± 12.9	0.60	−101.5 (−222.9, 19.9)
ACE III	88.4 ± 3.5	89.4 ± 3.6	0.20	−0.8 (−2.1, 0.5)
ICMR NCTB VLT total learning (TL) (T1 + T2 + T3)	20 ± 4.4	20 ± 3.3	0.70	0 (−1, 0)
ICMR NCTB VLT LOT	4 (2, 5)	4 (2, 4)	0.50	0 (−1, 0)
ICMR NCTB VLT delayed recall	7 (5, 8)	7 (5, 8)	0.30	0 (0, 2)
ICMR NCTB VLT delayed recognition	19 ± 2	19.6 ± 1	0.08	−0.9 (−1.9, 0.1)
ICMR NCTB TNI-93 total recall (free recall + cued recall)	8.5 ± 0.9	8.5 ± 0.8	0.50	−0.1 (−0.5, 0.3)
ICMR NCTB TNI-93 spatial recall	7.7 ± 1.7	8.1 ± 1.4	0.20	−0.3 (−0.9, 0.2)
ICMR NCTB trail making test part A	80 (70, 90)	85 (78, 93)	0.20	0 (−4.9, 14.9)
ICMR NCTB trail making test part B	162 (118, 206)	143 (127, 177)	0.40	0 (−80.7, 12.9)
ICMR NCTB CF vegetables	11.4 ± 4.1	11 ± 3.6	0.80	−0.1 (−0.9, 0.7)
ICMR NCTB CF animals	10.2 ± 2.6	10.8 ± 3	0.30	−0.6 (−1.7, 0.6)
ICMR NCTB CF food items	10.6 ± 5	9.7 ± 4.1	0.20	0.5 (−0.4, 1.4)
ICMR NCTB picture naming test	86.4 ± 4.7	87.4 ± 5	0.03	−1.5 (−2.9, −0.2)
Time taken to copy in stick design test (in seconds)	183.5 (139, 220)	173 (149, 240)	0.60	0 (−36.7, 15.1)
Score for copy	9.1 ± 1.5	8.6 ± 1.5	0.20	0.2 (−0.1, 0.6)
Time taken for immediate recall in stick design test (in seconds)	164 (132.5, 209.5)	157 (140, 218)	0.90	0 (−22.6, 25.4)
Score for immediate recall	8.4 ± 2.2	7.8 ± 2.1	0.09	0.5 (−0.1, 1.2)
Time taken for delayed recall in stick design test (in seconds)	166 (121, 216.5)	152 (141, 182)	0.90	0 (−24.8, 13.4)
Score for delayed recall	8.1 ± 2.5	7.6 ± 2.3	0.17	0.3 (−0.1, 0.3)
Stick design score – Total	25.6 ± 6.1	24 ± 5.4	0.10	1.1 (−0.3, 2.4)

#### Secondary outcomes

##### Feasibility

At central site, recruitment rate was 12.5%. Adherence of eight participants was assessed: ≥2 of the five intervention components was high, with full participation in cognitive activities (100%), physical activity (75%), and dietary changes (75%). Vascular risk monitoring was reported by 87.5% of participants. However, retention rate was lower (42.4%), attributable to lack of feasibility of participants travelling to central site. Therefore, neuropsychological assessments were not conducted but they were telephonically followed up for adherence.

At periphery sites, recruitment rate was higher with a six-month retention rate: 85% (3/20 dropouts). Adherence to ≥2 intervention components remained high, with 100% engagement in cognitive activities, 88.2% in physical activity, and 70.6% in dietary changes. Vascular risk monitoring was reported by 52.9% of participants. Among those with addictions, 62.5% reported reduction in dependence.

##### Preliminary intervention effects

At central site, all participants in intervention group (*n* = 8) achieved target MIND diet score (≥8) at six months, reflecting good adherence to dietary recommendations. 87.5% participants reported physical activity levels >600 METS per week, and 75% achieved BP control (<140/90 mmHg). Both participants who consumed alcohol at baseline attained optimal alcohol use scores (CAGEAID <1). No participants reported smoking at baseline. Furthermore, an improvement in cognitive scores was observed in both intervention and control arms (Table 2), but it was not statistically significant.

Participants at periphery sites also met target MIND diet score (≥8). 70.6% of participants achieved physical activity levels >600 METS per week, and 58.8% attained BP control. Among the target participants, none met the target alcohol use score (CAGE-AID <1), while out of six participants, scores of three participants remained same and scores of other three reduced. Also, there was an improvement in cognitive scores of some neuropsychological tests but no assessment except Picture Naming Test was found significant (Table 3).

Also, qualitative feedback indicated that understanding of booklet’s content was high at periphery sites and central site (≥80%). The motivation to use CBM was equally high: ≥87.5% indicating integration of recommended activities into daily practise. The willingness to continue the intervention after the six-month follow-up was 80–100% across periphery sites, indicating higher acceptability. No harm was reported by any participant during the intervention.

### CBM videos

CBM videos include ten videos of 4–8 min each, made in three languages (Appendix: p. 25). As suggested by behavioural science expert, few takeaways were added at end of each video to reinforce learning. 22.1% participants (*n* = 86) rated understandability ranging from 44.5% to 88.9% and others rated 100%, 3.5% (*n* = 86) participants rated actionability as 66.7% while others rated it as 100%. Additionally, qualitative feedback given by participants indicated higher engagement by illiterate participants in videos than booklet (Appendix: p. 17-18).

## Discussion

The multimodal approach in healthcare is a structured method comprising three to five evidence based interventions to prevent and manage health conditions. The current study aimed to construct, validate and pilot test a multimodal intervention for dementia prevention in high-risk Indian elderly. We found a high CVI for booklet and excellent actionability and understandability scores for both print and audio-visual materials. Moreover, CVI scores across all states consistently showed high values, indicating strong inter-regional agreement on the relevance and clarity of materials. Literature suggests that validating multimodal interventions using CVI helps ensure their relevance and quality, particularly among older adults (Mustafa Khalid et al., 2024; Polit et al., 2007). The scores on PEMAT are consistent with previous studies validating PEMAT as a reliable tool for assessing patient education materials (Kate et al., 2020; Rooha et al., 2023).

The results of pilot feasibility study assessing the feasibility and preliminary effects of CBM booklet showed a significant reduction in LIBRA scores in both community and hospital settings. These findings are consistent with prior multidomain intervention trials (Kivipelto et al., 2013), which indicate that programmes addressing physical activity, diet, cognitive engagement, and vascular risk control can produce short-term reductions in risk factors and potentially delay cognitive decline. However, there was no significant improvement in cognitive tests in both settings. While a multidomain intervention can be impactful, sample size of the study was not enough to detect any effect in neuropsychological scores. Moreover, the pilot feasibility study was conducted to understand preliminary effects of CBM.

The retention rate at central site was low (overall: 42.4%), highlighting logistic and behavioural challenges in sustaining continuity in hospital settings. These challenges included travelling to another state, extreme weather conditions, stressful hospital environment, and unresponsiveness. Additionally, as most participants were caregivers accompanying patients, follow-up visits were difficult. Despite operational barriers, CBM booklet was successfully delivered to participants. And the recruitment and retention rates were good in the periphery (85% retention at 6 months). This partial adherence reflects participants’ resilience and indicates practicality of delivering lifestyle interventions in community settings. The reduced numbers at follow-up also raises the possibility of nonresponse bias, as participants who were lost to follow up may differ (in characteristics) from those who completed the study. These issues will be addressed in the definitive multicentric trial through enhanced participant retention strategies and appropriate approaches for handling missing data. The existing literature supports primary and multidomain interventions to prevent dementia in at-risk populations (Park et al., 2020).

Audio-visual content may be more effective for elderly since they tend to prefer participating in activities involving audio-visual content. Video-assisted interventions can stimulate interest among elderly and strengthen their self-efficacy to learn, adapt, and transform their health behaviours (Paragas and Barcelo, 2019; Wischer et al., 2018). Few studies on determinants of adherence in the FINGER and Multidomain Approach for Preventing Alzheimer’s Disease (MAPT) reported that adherence decreased with increasing intervention complexity and intensity, indicating the need to balance the burden of participants (Kivipelto et al., 2013; Vellas et al., 2014). To address this limitation, multiple short animated videos have been created in this study.

Our intervention was designed to be of low cost and feasible for areas with limited resources, in the form of booklet and short animated videos to support scalability. The videos include storyline and characters highlighting the sociocultural context of rural Indian community settings, making the content relatable and easy to disseminate through existing rural healthcare system. Since our intervention used the existing healthcare system without setting up any additional infrastructure, it can be easily integrated into routine primary healthcare system. To further establish scalability and sustainability, we are planning a larger multicentric trial which will assess the efficacy of intervention at scale, and involve ASHA workers (already working in community as health workers) to support wider reach, better acceptability and long-term sustainability within the community.

The module in this study is practical, comprehensible, multidimensional, and easy to implement. As an integrated approach, this intervention allows adaptation to diverse cultural contexts, feasible delivery by primary health care workers, thereby enhancing its scalability across varied settings in India, particularly dementia care. This mode of intervention can ensure that resources are optimised to empower individuals and communities through psychoeducation, aiming for early intervention by targeting modifiable risk factors. A rigorous validation of module has been done, followed by piloting of feasibility, acceptability and understandability.

The major limitation of this approach is heavy reliance on psychoeducation and whether a less intensive approach may successfully achieve sustained behaviour modification. Another limitation is a higher attrition rate which can be explained by logistic and behavioural challenges as explained above. Pilot feasibility testing in our study was done in small numbers resulting in imprecise results. The reduced follow-up sample also limited the precision and generalizability of the findings, particularly for the hospital-based comparison. The follow up period was short, so long term adherence and sustainability of change cannot be deciphered from these results. Also, we used the original version of the LIBRA Index, which may limit its cultural relevance. We plan to culturally adapt and validate it in future studies. Nevertheless, this study can provide important insights into challenges of behaviour modification in a diverse country like India and help tailor successful protocols for the prevention of dementia that are scalable.

## Conclusion

To conclude, the multimodal Care Bundle Module presents a comprehensive, structured, and evidence-based approach for dementia prevention in India. It offers a strategic, standardised intervention aimed at balancing the complexity and intensity of behaviour modification.

## Data Availability

The raw data supporting the conclusions of this article will be made available by the authors, without undue reservation.
