# Supplementary material for: Development of a multimodal health education module for dementia prevention in at-risk rural Indian elders: a SMRUTHI INDIA initiative
Source: Front Dement. 2026 Oct 1;5:1914082. doi: 10.3389/frdem.2026.1914082 (PMC13668599; doi:10.3389/frdem.2026.1914082)

Strategic Multimodal Intervention in at-risk Elderly Indians for Prevention of Dementia – A Cohort Multiple Randomized Controlled Trial (cmRCT)

SMRUTHI
INDIA

Care Bundle Module

A draft module developed by Team SMRUTHI-INDIA, AIIMS, New Delhi

For

Pilot testing in a study of primary prevention of dementia in elder Indians

Acknowledgements

We are grateful to the panel of experts who guided our team in the content development of this *Care Bundle Module* booklet.

What is Dementia?

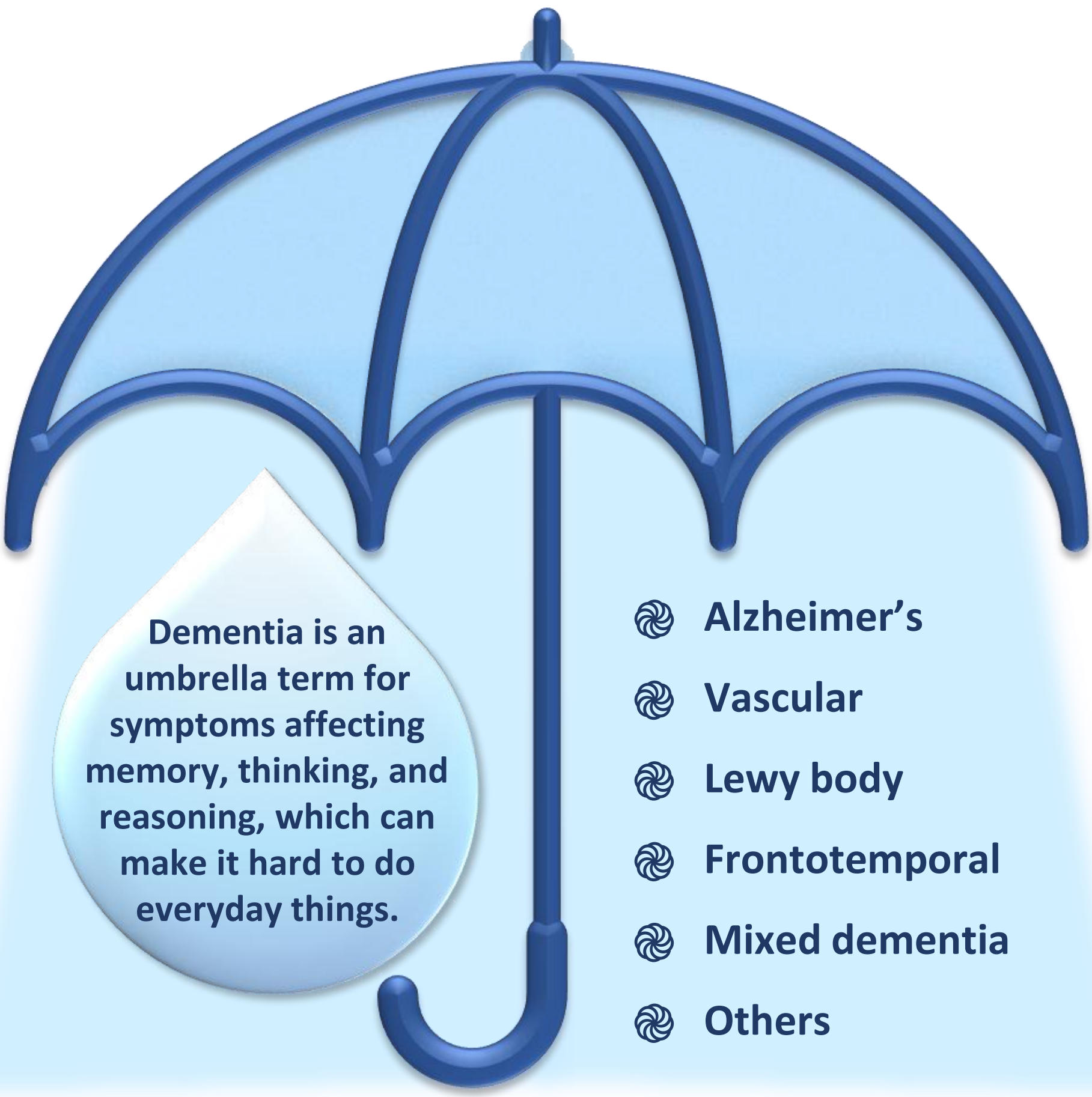

Supplement: Supplementary file 1 [file Supplementary_file_1.pdf]
