# Supplementary material for: Development of a multimodal health education module for dementia prevention in at-risk rural Indian elders: a SMRUTHI INDIA initiative
Source: Front Dement. 2026 Oct 1;5:1914082. doi: 10.3389/frdem.2026.1914082 (PMC13668599; doi:10.3389/frdem.2026.1914082)
Supplement: Supplementary file 2 [file Data_Sheet_1.docx]

**SUPPLEMENTARY APPENDIX**

| **S. No.** | **Table of Content** | **Page No.** |
| --- | --- | --- |
| 1 | Global studies on primary prevention of dementia | 2-10 |
| 2 | References | 11-12 |
| 3 | Items presented to experts for the content validity index | 13-14 |
| 4 | Disagreement of experts (Care Bundle Module Booklet) | 15 |
| 5 | Qualitative feedback by participants (CBM Booklet) | 16 |
| 6 | Qualitative feedback by participants (CBM Videos) | 17-18 |
| 7 | Post Intervention Adherence Scale (PIAS) | 19-21 |
| 8 | Tables | 22-25 |
| 9 | Proposed lifestyle intervention model for dementia prevention in at-risk elderly | 26 |
| 10 | Summary and agreement of responses from five experts | 27 |
| 11 | LIBRA Index | 28 |
| 12 | CONSORT Checklist | 29-33 |

**Global studies on primary prevention of dementia**

| **Sl. No.** | **Country and year** | **Design of study and sample size** | **Intervention type** | **Outcome assessed** | **Result** |
| --- | --- | --- | --- | --- | --- |
| 1. | FINGER  (Finnish Geriatric Intervention Study to Prevent Cognitive Impairment and Disability)^1^  2009-2014  currently extended follow up | 2-year Randomised Control Multi-Centre Double-blind Trial  (N)1260 older adults | **Control group**  Regular health care advice  **Experimental group**  Intensive Multimodal Intervention   - Nutritional guidance based on Finnish Nutrition Recommendation - Exercise (muscle strength training and aerobic exercises) (based on a modified version of Dose-Response to Exercise Training Protocol) - Cognitive training (group and individual sessions) using computer-based programs and social activities - Management of Metabolic and Vascular Risk factors | **Primary Outcome:**  Cognitive performance assessed by modified Neuropsychological test battery (mNTB).  **Secondary outcome**:  Incidence of dementia and Alzheimer’s Dementia, Cognition evaluation with mNTB domain z scores, Vascular risk factors, Cardiovascular and cerebrovascular morbidity and mortality, Dietary Intake and markers, Disability, Falls, Cardiorespiratory fitness, Depressive symptoms and Quality of Life | 1. Improved global cognition was observed after 24 months of intervention as assessed by the Neuropsychological Test Battery. (25% higher in intervention group than in control group) 2. Enhanced performance in cognitive sub-domains such as executive function and processing speed. (83% and 150% higher in intervention group compared to control group respectively). |
| 2. | U.S.POINTER  (The U.S. Study to Protect Brain Health through Lifestyle Intervention to reduce Risk)^2^  2019 – Present | 2-year multisite Randomized controlled trial  ( N) 2000 at risk elderly people | **Control group -**  Self-guided Lifestyle Intervention: Lifestyle health education and support  **Experimental group -**  Multimodal Intervention:   - Nutritional coaching (Regular team meetings and telephones) - Physical exercise (group exercise) - Cognitive exercise (computer cognitive training, team meetings) - Guideline-based health monitoring every 6 months | **Primary Outcome**  Global Cognition composite score (POINTER modified Neuropsychological test battery PmNTB )  **Secondary Outcome**  Cognitive Domain-specific composite score (Memory, Executive function and Processing speed)  **Exploratory Measures**  Clinical and behavioural outcomes (score  on Clinician Dementia rating Scale and self-report questionnaire. | 1. Both groups improved cognitively, with the structured lifestyle program showing stronger gains. 2. A multi-domain lifestyle approach (exercise, diet, engagement, health monitoring) helps protect brain health in at- risk older adults. |
| 3. | SINGER (The  SINgapore GERiatric Intervention Study to Reduce Cognitive Decline and Physical Frailty Study Design and Protocol)^3^  (2022-present) | 2 Year, Multisite Randomised control trial  (N)1200 older adults at risk | **Control group-**  Self-guided Lifestyle Intervention : Health education, support and care through guideline based health monitoring, supportive tools, physical examination and blood laboratory tests.  **Experimental group –**  Structured Lifestyle Intervention (with goal setting):   - Vascular Management – based on government health guideline - Cognitive training – Cognitive stimulation through workshops and computer-based cognitive training program - Physical training – muscle training and aerobic training - Nutritional counselling - Individual and group session | **Primary Outcome:**  Global cognitive performance (composite z score of Neuropsychological battery)  **Secondary Outcome**  Effect of intervention on specific Cognitive domains, Functional abilities, Lifestyle factors, Vascular risk factors (change in Blood Pressure, lipid profile, glucose), Neuroimaging (Cerebrovascular disease markers, MRI, vascular polygenic risk scores), Blood biomarkers, Retinal imaging and Cost-benefit analysis | Study in progress |

| **Sl. No.** | **Country and year** | **Design of study and sample size** | **Intervention type** | **Outcome assessed** | **Result** |
| --- | --- | --- | --- | --- | --- |
| 4. | SINGER 6-month Pilot Study (The SINgapore GERiatric Intervention Study to Reduce Cognitive Decline and Physical Frailty)^4^  2019-2020 | 6 month Randomised control trial  (N) 70 elderly participants | **Control group**  **FINGER** Cognitive training- Computerised mode; Diet- Nordic/Mediterranean diet, Physical exercise – unsupervised aerobic and supervised strength sessions, Vascular risk factor management  **Experimental group SINGER**  Cognitive training – Pen and paper mode; Diet - Local Asian diet, Physical exercise – supervised aerobic and strength sessions, Vascular risk factor management | **Outcome Measures**  **Feasibility** (Diet, Vascular monitoring, Cognitive training, Exercise)  **Efficacy** (Diet and Vascular Management, Cognitive Intervention and Physical Intervention) | 1. Both interventions equally feasible with participants completing 80% of the intervention 2. SINGER cognitive training provided greater feasibility and SINGER group attained good blood pressure control at 6 month compared to FINGER 3. Multidomain lifestyle intervention has positive effect on mild/moderate older adults. |
| 5 | AgeWell.de Trial, Germany^5^  2018-2020 | 2-year Multicentric Cluster Randomised Control Prevention Trial  (N) 1152  participants | **Control Group**:  General health advice and treatment as usual  **Experimental Group** -   - Nutritional counseling – based on government guidelines, - Enhancement of physical activity and social engagement, - Cognitive training – with computer tablets and software - Optimization of medication - Management of vascular risk factors (overweight, smoking), - Intervention of bereavement, grief, and depressive symptoms - Motivational task across all components | **Primary outcome:**  Change in the cognitive performance (assessed by neuropsychological test battery)  **Secondary outcome:**  Mortality, Nursing home placement, Functioning in everyday activities, Quality of life , Mood/depressive symptoms, Social inclusion  Resource utilization and costs | 1. No significant intervention effect on the global cognitive performance 2. Intervention improved health related quality of life and reduced depressive symptoms in women 3. COVID-19 Pandemic imposed challenges in conducting intervention |
| 6. | AU-ARROW  (AUstralian multidomain Approach to Reduce dementia Risk by prOtecting brain health With lifestyle intervention Study)^6^  2020 – present | 2 years Multimodal Intervention  (N) 600 participants | **Control group:**  Health education coaching group - attending information, 5 group meetings in 2 years, discussing diet, exercise, cognitive activities and goal setting with medical counseling  **Experimental group -**  Structured Multi Domain Lifestyle Intervention group:   - Diet – Diet education, introduce MIND diet - Physical exercise – aerobic exercise, resistance, stretching, and balance training - Cognitive training – computer-based training from Brain HQ- web based program - General medical health education - Monthly meeting | **Primary Outcome:**  Global composite cognitive Score (based on AU-ARROW modified Neuropsychological test battery)  **Secondary outcome**  Effect of intervention on specific cognitive domains, cardiovascular risk factors, safety, and feasibility of intervention, genetic risk (Apolipoprotein E)  **Exploratory Outcome:**  Impact of intervention on hippocampal volume and brain AB amyloid over 2 years  Explore the retinal imaging, and identify  blood and urine biomarkers for early detection of Alzheimer's disease | Study in progress |

| **Sl. No.** | **Country and year** | **Design of study and sample size** | **Intervention type** | **Outcome assessed** | **Result** |
| --- | --- | --- | --- | --- | --- |
| 7. | MYB AUSTRALIA  (Maintain Your Brain – Australia)^7^  2018 – Present | 3 years online single- blind randomized controlled trial of personalized multimodal intervention  (N) 8500 adults aged between 55 and 75 years | **Control group:**  Information: Receive monthly information and period reminder emails  **Experimental group:**   - Nutrition (Mediterranean diet through messages, feedback, and materials) - Physical Activity – aerobic exercise, resistance and balance training. (through regular feedback and action plan and general progression from moderate to vigorous training) - Peace of Mind (Mixed anxiety and depression course through psychotherapy, cognitive therapy, thought challenging, advanced graded exposure, and relapse prevention) - Brain training (assess baseline cognitive domain like verbal executive speed, visual memory, visual attention) (For adherence and motivation: several socialization and gaming strategies through MYB digital platform. | **Primary Outcome:**  Change in cognition from baseline to three years (measured by MYB online cognitive test Battery)  **Secondary outcomes**  Cognitive impairment and dementia: Dementia incidence at 3 years, change in risk of dementia, change in specific cognitive domains  Overall impact and participation experience: utilization of service, cost of the program, altered expectations, compliance, record of adverse events  Risk factors related : to change in BMI, Physical activity, physical functional performance, new chronic condition, adherence to Mediterranean diet, change in alcohol and smoking status, mental activity level, and psychological distress. | 1. Online multidomain lifestyle training improved cognition and participants showed ~25% lower risk of cognitive decline compared with controls. 2. High-engagement users had significantly greater cognitive gains across memory, attention, and executive function. |
| 8. | MIND CHINA  (Multidomain INtervention to delay Dementia and disability in rural China)^8^  2018- present | Ongoing cluster Randomised controlled Multimodal Intervention  (N)5765 participants | **Control Group:**  Standard of care: Regular healthcare services  **Experimental Group:**  *Vascular intervention group* –   - Health education - Medical treatment aimed to control the risk factors (hypertension, diabetes, and dyslipidemia)   *Multimodal intervention -*   - Health education - Medical treatment aimed to control the risk factors (hypertension, diabetes, and dyslipidemia) - Lifestyle and dietary guidance - Group exercise - Personalized leisure activities - Cognitive training | **Baseline data provided:**  Epidemiology of dementia and cognitive Aging  Risk and protective factors for dementia Clinical and imaging markers for  Alzheimer’s dementia and dementia  To understand the natural history of cognitive aging and effect of multimodal intervention | Interim Analysis:  Baseline Assessment found mean age of population as 70.9 years (SD 5.9) with 57.2% women, 40.6% illiterate and  88.3% were farmers.  Overall prevalence was found to be: 67.2% for hypertension,  23.4% for dyslipidemia  23.5Z% for heart disease 14.4% for diabetes mellitus 5.4% for dementia  Prevalence of hypertension, diabetes mellitus, obesity, heart disease, depressive symptoms and dementia higher in women (P < 0.05). |

| **Sl. No.** | **Country and year** | **Design of study and sample size** | **Intervention type** | **Outcome assessed** | **Result** |
| --- | --- | --- | --- | --- | --- |
| 9 | PENSA  (Prevention of Cognitive Decline in Subjective cognitive decline APOEe4 carriers after EGCG (Epigallocatechin gallate) and Multimodal Intervention, Spain)^9^  2019 – Present | 12 months Randomised double- blind, personalized, controlled clinical trial  (N) 200 participants with  *APOE* ε4 *carriers* with Subjective cognitive decline (either hetero or homozygotes) | **Control group -**   1. Lifestyle Recommendations + EGCG (Epigallocatechin gallate) dietary supplement (400 to 600 mg prior to meals)   (N 50)   2. Lifestyle Recommendation + Placebo (N 50)   **Experimental group-**   3. Multimodal Intervention    - Personalized diet based on MedDiet,    - Physical activity- aerobic, strength and balance activities guided by physiotherapist    - Cognitive training through web-based platform NeuronUP    - Psychoeducation, social stimulation activities) via digital platform + EGCG (Epigallocatechin gallate) 4. Multimodal Intervention (as mentioned above) + Placebo | **Primary Outcome:**  Change in global cognition (using Neuropsychological tests)  **Secondary Outcome:**  Change in brain connectivity (MRI and f MRI)  Safety assessment of EGCG through blood samples assessing thyroid, liver and kidney function  **Exploratory outcomes** Biomarker analysis :  Blood - assess safety parameters, AD- related biomarkers, microbiota-related and compliance biomarkers, plasma;  urine and faeces - assess metabolomics,  dietary profiling, and related metabolites | 1. At 12 months, no significant cognitive difference was found between EGCG + multimodal intervention vs. placebo (mean difference = 0.12; 95% CI: –0.01   to 0.24; p = 0.061).   2. After a 3-month wash-out, EGCG showed significant cognitive benefit, outperforming placebo (mean difference = 0.19; 95% CI: 0.06–0.32; p = 0.005). |
| 10 | J-MINT,  (Japan - Multimodal Intervention Trial for Prevention of Dementia)^10^  2019-Present | 18 month, open labelled, randomised, controlled, multicentre trial of multidomain intervention  (N)500 participants | **Control group -**  Health-related information in every 2 months, management of vascular risk factors  **Experimental Group:**  **Intensive Intervention group-**   - Nutritional Counselling – based on Dietary Reference Index for Japanese; - Group-based physical exercise and self-monitoring of physical   activity- (including dual task exercise: physical and cognitive task called  ‘cognicise’)   - Cognitive training using the web-based program Brain HQ (Japanese version) - Management of vascular risk factors with the aid of government-based guidelines | **Primary Outcome:**  Cognitive Change at 18 month follow up (composite score)(assessed by neuropsychological tests)  **Secondary Outcome:**  Change in cognitive domain: based on comprehensive geriatric assessment , Blood and Urinary test:  Glucose, insulin, lipid profile, liver and kidney function test, thyroid hormones, vitamins, inflammatory markers and APOE phenotype.  Blood based dementia related biomarkers and whole genomic sequencing  Brain MRI and CT scans  Adverse and serious adverse events | 1. Overall effect was small and not statistically significant: Global cognition improved only 0.047 points vs control (95% CI −0.029 to 0.124). 2. But certain subgroups showed meaningful memory gains: APOE-ε4 carriers and high-adherence participants had significant improvement in logical memory (p < 0.05). Study in progress |
| 11 | CAN - THUMBS UP  (The Canadian Therapeutic Platform Trial for Multidomain Intervention to Prevent Dementia, Canada)^11^ | Prospective 12 month multicentre longitudinal study One group pretest post-test design (N)350 Participants who are:  Cognitively  Unimpaired, Subjective Cognitive | **Experimental Group:**  BHPro, a 45-week, web-based formal education program (8 modules and 181 chapters) for aiding Lifestyle Intervention  Program content- General Information on Cognition, Dementia, genetic risk and healthy lifestyle, Physical activity, cognitive engagement, diet, sleep , social and psychological health, Vascular health, Vision and hearing  Personalised profile on modifiable lifestyle risk factors  Based on responses, alongside setting and revisiting goals aligned with their risk profile throughout the study | **Primary Outcome:**  Change in dementia literacy  **Secondary Outcome:**  Self-efficacy, engagement using online program, user satisfaction rating, evaluation of usability and acceptance  **Exploratory Outcome:** | Study in progress |

| **Sl. No.** | **Country and year** | **Design of study and sample size** | **Intervention type** | **Outcome assessed** | **Result** |
| --- | --- | --- | --- | --- | --- |
|  | Prospective Study | Impairment and Mild Cognitive Impairment |  | Change in attitude towards dementia and screening, change in risk factors, cognitive  domains, physical activity and sleep quality |  |
| 12 | SUPERBRAIN  (So**U**th Korean study to **P**r**E**vent cognitive impai**R**ment and protect **BRAIN** health through lifestyle intervention in at- risk Elderly population)^12^ 2019- Present | Multicentre, outcome assessor blinded, Randomised, Controlled Feasibility Trial  (N)152 participants with age range of 60-79 years | **Control Group:**  Consult General physician, receive Booklet on lifestyle guidance to prevent dementia  **Experimental Group (2 groups):**  A. Facility-based Multidomain Intervention (FMI) Multidomain Intervention is of 24 weeks  Intervention applied in both experimental groups:   - Nutritional guidance (3 individual and 7 group sessions using MIND diet - Monitoring and management of metabolic and vascular risk factors - Cognitive training targeting various domains with application or workbook- based intervention, along with social activity - Physical exercise (aerobic exercise, muscle strengthening, enhance balance and flexibility) - Motivational training (4 group counseling sessions)   B. Home-based Multidomain Intervention (HMI) - (frequent group and home sessions)  Management for metabolic and vascular risk factors ,social activity and motivational enhancement programs are identical with FMI.  Group and home-based activities for cognitive training and physical exercise. | **Outcome Measure**  Adherence , Retention rate , and Change in total scale index score of the repeatable battery for assessment of neuropsychological status (RBANS)  **Secondary outcomes**:  Effect on Cognition, Mood Disability, Quality of Life, Physical Function, Nutrition,  Vascular risk, Motivation Sleep and Progression  **Exploratory Outcome:** Electroencephalography, Brain MRI, Actigraphy, Telomere length, Neurotrophic factor, Neurodegenerative factor, Plasma amyloid – B 42, Microbiome | 1. After 24 weeks, the multidomain intervention group showed a +8.43 increase in RBANS global cognition vs +4.26 in controls (difference   +4.17, 95% CI 1.92–6.43, p <  0.001).   2. Participants maintained ~85% adherence, showing the multidomain lifestyle program is practical and well-accepted in at-risk older adults. |
| 13 | MIND – AD  (Multimodal Preventive Trial for Alzheimer’s Disease , Germany, France, Sweden, Finland)^13^ 2017- present (delay due to Covid-19) | 6-month multinational proof of concept Randomised Controlled Trial  (N)93 participants with prodromal AD | **Control** group – Regular Health Advice/Care  **Experimental Group (2 groups)** – 1 Multimodal Lifestyle Intervention –   - Nutritional guidance (followed New Nordic Recommendations or country’s   nutritional guidance)   - Physical exercise (Group sessions and training based on FINGER trial and ADEX trial) - Cognitive training (computer-based training, adapted from FINGER trial) - Intensive monitoring and management of vascular risk factors (blood pressure, weight, BMI) - Social stimulation (group meetings)   2.Multimodal Lifestyle intervention along with Medical food (Fortasyn Connect) Intervention group:  It included all of the above and the study product Fortasyn Connect, 125 ml once a day, mild-based drink.  Fortasyn Connect is a multi-nutrient combination designed to enhance efficacy | **Primary Outcome:**  Recruitment rate, Overall  Adherence to intervention and Retention rate  **Secondary Outcome:**  Adherence to intervention components and to healthy lifestyle changes  **Exploratory Outcome:**  Changes in vascular and metabolic risk factors, depressive symptoms, stress- related symptoms, physical performance, health-related quality of life | 1. The 6-month multidomain lifestyle intervention (with or without nutritional supplement) showed high adherence (~75–80%) and was safe, meeting feasibility criteria for larger trials. 2. Participants in the intervention arms showed improvements in functional ability and some cognitive measures compared with usual care, but the study was not powered for statistical significance. |
| 14 | GOIZ ZANDU  trial: a FINGER- like multidomain | One-year controlled, randomized, | **Control group:** | **Primary Outcome:**  Feasibility - Adherence to intervention and Retention rate | Retention rate was found to be 88 percent in the intervention group. |

| **Sl. No.** | **Country and year** | **Design of study and sample size** | **Intervention type** | **Outcome assessed** | **Result** |
| --- | --- | --- | --- | --- | --- |
|  | lifestyle intervention feasibility study to prevent dementia in Southern Europe  (Basque, Spain)^14^ (2018-2020) | Multidomain Intervention trial  125 (N) participants | Regular Health Advice Control (RHA) group - Provide preventive programs at the Primary Care Unit regarding physical activity, socialization, smoking, and alcohol usage.  **Experimental group:**  Multidomain Intervention (MD-Int) group:   - Intensive monitoring and management of vascular risk factors (3-month follow-up) - Nutritional counseling (Mediterranean diet pattern through workshop, verbal and written material) - Physical activity and exercise (based on American Heart Association Guidelines and the ICOPE – Integrated Care for Older People by WHO) - Cognitive intervention – to introduce cognitively stimulating daily habits   and routine with importance to family and social setting) | **Efficacy exploratory outcomes**: Difference between two groups in a Global Cognition composite (LatAm- Neuropsychological Test Battery) Safety Assessment:  Metabolic and vascular risk factors through blood count, cholesterol, glucose, renal and liver function, thyroid hormones, B12 vitamin, folic acid | Adherence to the Mediterranean diet and physical fitness were found to be slightly higher in the intervention group (p<0.05).  Overall adherence to all intervention activities was “Good” in 71.9% of the participants.  There was a significant difference between the scores of the two groups in the Executive test of Neuropsychological test battery |
| 15 | LatAm-FINGERS  (Latin American Initiative for Lifestyle Intervention to Prevent Cognitive Decline )  (14 Latin American countries)^15^  (2021 – Present) | One-year randomized controlled lifestyle intervention trial  (N) 815 randomised  Aged 60 and 77 years | **Control group:**  Flexible Lifestyle Intervention:  Regular Medical advice which included one meeting that reviews the recommendation of health care based in the guidelines  **Experimental group:**  Systematic Lifestyle Intervention:   - Physical exercise: Exercises were in phases such as warm-up, coordination, stretching, and balance; resistance and muscle strength training, aerobic exercise and relaxation activities. - Nutritional intervention: Formulated a ‘mosaic’ program to provide 16 different alternate meals within the LatAm-MIND diet - Cognitive Intervention: Educational meetings and computerized regular cognitive training catering to various domains - Medical monitoring intervention: Medical interventions were carried out by a general practitioner, family doctor, geriatrician, or neurologist. | **Primary Outcome:**  Cognitive domain based on LatAm- Neuropsychological tests.  Feasibility of intervention based on Re- AIM (Reach, Efficacy, Adoption, Implementation and Maintenance)  Efficacy – Changes in diet, physical activity and health metric  **Secondary Outcome:** Cognitive Processing speed,  Cognitive memory,  Execution, Cognitive Global functional, Lifestyle  **Exploratory measures:** Cardiovascular risk, sleep habits, metabolic, mood, Quality of life, and physical performance. | Interim Analysis:  Participants are ethnically diverse and have high prevalence of metabolic syndrome (39 percent).  Over representation of Women in sample across various centres  (64% in Mexico and 85% in Colombia) leading to discrepancy in sex recruitment. |
| 16 | PreDIVA  (Prevention of Dementia by Intensive Vascular Care: A Cluster – randomized Netherlands)^16^  (2006-2014) | Multisite, open, cluster-randomised, parallel-group study  Large Cluster Randomised Trial  (N)3526 elderly subjects (70-78 years of age) | **Control Group:**  Standard Care Condition – Care according to Dutch general practice  **Experimental Group:**  Intensive Vascular Care Condition –   - Exercise – assessment of current level and individualized advice regarding physical activity - Smoking – Assessment of smoking status and readiness to quit (if required nicotine replacement therapy, behavioral advice, and counseling) - Weight- BMI>25 were given behavioural and nutritional counseling | **Primary outcome:**  Cumulative Incidence of dementia and Disability Score at 6 years follow up  **Secondary outcome:** Mortality, vascular event incidence, effect on mood, cognitive functions like Memory task sensitive to early stages of AD. | No effect of 6.7 years of intensive vascular care on incident all-cause dementia, disability or mortality dementia. |

| **Sl. No.** | **Country and year** | **Design of study and sample size** | **Intervention type** | **Outcome assessed** | **Result** |
| --- | --- | --- | --- | --- | --- |
|  |  |  | - Blood pressure – Lifestyle modifications like exercise, weight, sodium restriction, moderation of alcohol , cessation of smoking) - Blood lipids: subjects with known cardiovascular disease: statin (eg simvastin 40 mg) - Blood Glucose and Cardiovascular history |  |  |
| 17 | A Multidomain approach for preventing Alzheimer’s disease (MAPT Study)^17^ | Multicentered, randomized, placebo- controlled trial among at-risk population of cognitive decline  (N) = 1680  participants aged 70 and above living in the community and at risk of cognitive decline | **Control group** –  Placebo group – Consume 2 placebo capsules daily for 3 years  **Experimental group** –  Omega dose only – Participants have to consume 800 mg of docosahexaenoic acid per day ( 2 soft capsules daily) as a single dose  Multidomain intervention only –   - Nutrition – Based on dietary guidelines of the French National Nutrition and Health Program. - Physical activity – Participants were encouraged to perform moderately intensive physical like aerobic or strength training activities along with a personalized home-based physical activity program - Cognitive Training – Initial sessions focused on Reasoning Training and identifying the next item in the series. Later sessions focused on memory Training   Omega 3 (800mg DHA per day) and multidomain intervention – Components of Multidomain intervention and Omega 3 were administered to the participants | **Primary Outcome:**  Change in memory function after 3 years follow up and Progress in memory decline  **Secondary Outcome:**  Efficacy of intervention on Cognitive performances  lexical and categorical fluency , Physical function test, assess Frailty Syndrome , Depression ,  Tolerability to Omega 3 , Adherence to Intervention ,  Cost-effectiveness of intervention  **Exploratory outcome:**  Ancillary studies assessed neuroimaging to explore the effect of intervention on cerebral atrophy rate(MRI Scans), cerebral metabolism (FDG-PET Scan) and amyloid deposit (florbetapir PET Scans) | Interim Analysis: Preliminary Result:  Spontaneous memory complaints were present in 99.1% of participants (treatment and placebo group)  11.2% had limitations in at least one IADL  11.9% reported slow walking speed at baseline  42% of participants had CDR equal to 0 and 0.5  42.1% of participants were considered pre-frail and 3.2% were frail  Neuroimaging results are in progress. |
| 18 | Multidomain Intervention for Reversal of Cognitive Frailty, Towards a Personalised Approach (AGELESS Trial),  Malaysia^18^ 2020 - present | 2-year intervention study Multicentre, longitudinal, double- blind randomized controlled trial  (N) 330 older adults from urban, semi- urban, and rural areas | **Phases of study:**  Phase 1 : Development of Intervention Package (Nutritional Intervention, Physical activity, Cognitive training, Psychosocial guidance, Management of metabolic and vascular risk factors) followed by training of trainers  Phase 2 : Screening of cognitively frail older adults, Baseline Assessment, and Randomisation of (N 330) participants at 3 centers (urban, semi-urban and rural) with baseline assessment at 6, 12, and 24 months  Phase 3: Implementation of the study:  **Control group –** Routine care and general health consultation such as blood pressure, anthropometry measurements, receive group counseling and standard public health pamphlet  **Experimental group –**   - Nutritional Counseling- Nutritional guidance based on Malaysian Dietary Guidelines for Older Adults and Malaysian Recommended Nutrient Intake for individuals aged 60 and above. | **Primary Outcome:**  Cognitive Frailty Status/Cognitive Function – (assessed through Modified Neuropsychological Test Battery) Physical Fitness/Function  **Secondary Outcome:**  Effect on Body Composition, Psychosocial and functional status, Dietary Intake,  Vascular Health, assess Faecal sample and Brain Activation | Cognitive frailty status and cognitive function improved significantly in the multidomain-intervention group (Modified Neuropsychological Test Battery scores ↑; group × time p < 0.05).  Physical fitness/function also improved (e.g., gait speed, balance, strength; all p < 0.05).  Body composition, vascular health, diet quality, and psychosocial wellbeing showed significant improvement (p < 0.05).  Improvements were also observed in functional status, with additional exploratory gains in faecal microbiome  markers and brain activation patterns. |

| **Sl.**  **No.** | **Country and year** | **Design of study and sample size** | **Intervention type** | **Outcome assessed** | **Resu lt** |
| --- | --- | --- | --- | --- | --- |
|  |  |  | - Psychosocial Intervention – Transtheoretical model of change was implemented. - Physical Activity – Modified version of Dose Responses to Exercise Training focused on strength, aerobic, postural balance, and stretching training. - Cognitive training – guided practice to train global cognition and its domains along with culturally adapted training module - Metabolic and Vascular Risk Factors Management   Phase 3: Modelling Personalised Rehabilitation Approach to Reduce risk of Cognitive frailty. Further assess the feasibility and implementation in the urban  and semi-urban area. |  |  |
| 19 | The Taiwan Health promotion Intervention Studyfor Community Elders (THISCE)^19^  (2014-2017) | 12-month prospective cluster randomised controlled trial  Efficacy study (N)1082  Empowerment Study  (N) 440  Participants were 65years or older havingat least 3 chronic medical conditions | - **Efficacy Study: Control Group**: Usual health care   **Experimental Group**: Standard Multidomain Intervention Assess the efficacy of 12-month participatory community- group multidomainintervention against physical and cognitive decline among prefrail/frail community-dwelling older people   - **Empowerment Study :Experimental groups:** - Standard Multidomain Intervention - Enhanced Multidomain Intervention – training sessions to use newteaching material, revised and simplified from the standard multidomain prototype.   Evaluating the benefits of empowering the individual participants by replicating the format of the efficacy study. | **Primary Outcome:**  Changes from baseline in CHS Frailty score, gait speed, grip strength, and adjusted Montreal Cognitive Assessment (MoCAadj. )  **Secondary Outcome**: Instrumental Activities of Daily Living, Nutrition status, and Depressive symptom | Efficacy Study:  6 months later, no significant improvement in overall physical or functional outcome however, concentration improved significantly.  12 months later, the intervention groupwas less likely to have depressed mood, malnourished, and enhanced concentration.  Empowerment Study:  Results showed similarities to efficacy study with enhanced gait speed, physicalactivity, lower malnutrition prevalence, and improved cognitive performance butno significant  differences in depressed mood between intervention groups. |
|  |  |  | MRI – Magnetic Resonating ImagingCT -Computed Tomography Scan   - FDG-PET – Fludeoxyglucose18 positron emission tomography |  |  |

**References**

1. Kivipelto M, Solomon A, Ahtiluoto S, Ngandu T, Lehtisalo J, Antikainen R, et al. The Finnish Geriatric Intervention Study to Prevent Cognitive Impairment and Disability (FINGER): Study design and progress. Alzheimer’s & Dementia. 2013 Nov 1;9(6):657–65.
2. Baker LD, Snyder HM, Espeland MA, Whitmer RA, Kivipelto M, Woolard N, et al. Study design and methods: U.S. study to protect brain health through lifestyle intervention to reduce risk (U.S. POINTER). Alzheimer’s & Dementia. 2024;20(2):769–82.
3. Xu X, Chew KA, Wong ZX, Phua AKS, Chong EJY, Teo CKL, et al. The SINgapore GERiatric Intervention Study to Reduce Cognitive Decline and Physical Frailty (SINGER): Study Design and Protocol. J Prev Alzheimers Dis. 2022 Jan 1;9(1):40–8.
4. Chew KA, Xu X, Siongco P, Villaraza S, Phua AKS, Wong ZX, et al. SINgapore GERiatric intervention study to reduce physical frailty and cognitive decline (SINGER)–pilot: A feasibility study. Alzheimer’s & Dementia: Translational Research & Clinical Interventions. 2021;7(1):e12141.
5. AgeWell.de – study protocol of a pragmatic multi-center cluster-randomized controlled prevention trial against cognitive decline in older primary care patients | BMC Geriatrics [Internet]. [cited 2025 Dec 12]. Available from: https://link.springer.com/article/10.1186/s12877-019-1212-1
6. Gardener SL, Fuller SJ, Naismith SL, Baker L, Kivipelto M, Villemagne VL, et al. The AUstralian multidomain Approach to Reduce dementia Risk by prOtecting brain health With lifestyle intervention study (AU-ARROW): A study protocol for a single-blind, multi-site, randomized controlled trial. Alzheimer’s & Dementia: Translational Research & Clinical Interventions. 2024;10(2):e12466.
7. Maintain Your Brain: Protocol of a 3-Year Randomized Controlled Trial of a Personalized Multi-Modal Digital Health Intervention to Prevent Cognitive Decline Among Community Dwelling 55 to 77 Year Olds

- Megan Heffernan, Gavin Andrews, Maria A. Fiatarone Singh, Michael Valenzuela, Kaarin J. Anstey, Anthony J. Maeder, John McNeil, Louisa Jorm, Nicola T. Lautenschlager, Perminder S. Sachdev, Jeewani

A. Ginige, Megan J. Hobbs, Christos Boulamatsis, Tiffany Chau, Lynne Cobiac, Kay L. Cox, Kenneth Daniel, Victoria M. Flood, Yareni Guerrero, Jane Gunn, Nidhi Jain, Nicole A. Kochan, Amit Lampit, Yorgi Mavros, Jacinda Meiklejohn, Yian Noble, Fiona O’Leary, Sue Radd-Vagenas, Courtney C. Walton, , Maintain Your Brain Collaborative Team, Maintain Your Brain Collaborative Team, Henry Brodaty, 2019 [Internet]. [cited 2025 Dec 12]. Available from: https://journals.sagepub.com/doi/full/10.3233/JAD-180572

8. Wang Y, Han X, Zhang X, Zhang Z, Cong L, Tang S, et al. Health status and risk profiles for brain aging of rural-dwelling older adults: Data from the interdisciplinary baseline assessments in MIND-China. Alzheimer’s & Dementia: Translational Research & Clinical Interventions. 2022;8(1):e12254.
9. Forcano L, Fauria K, Soldevila-Domenech N, Minguillón C, Lorenzo T, Cuenca-Royo A, et al. Prevention of cognitive decline in subjective cognitive decline APOE ε4 carriers after EGCG and a multimodal intervention (PENSA): Study design. Alzheimer’s & Dementia: Translational Research & Clinical Interventions. 2021;7(1):e12155.
10. Sakurai T, Sugimoto T, Akatsu H, Doi T, Fujiwara Y, Hirakawa A, et al. Japan-Multimodal Intervention Trial for the Prevention of Dementia: A randomized controlled trial. Alzheimer’s & Dementia. 2024;20(6):3918–30.
11. Feldman HH, Belleville S, Nygaard HB, Montero-Odasso M, Durant J, Lupo JL, et al. Protocol for the Brain Health Support Program Study of the Canadian Therapeutic Platform Trial for Multidomain Interventions to Prevent Dementia (CAN-THUMBS UP): A Prospective 12-Month Intervention Study. J Prev Alzheimers Dis. 2023 Nov 1;10(4):875–85.
12. Park HK, Jeong JH, Moon SY, Park YK, Hong CH, Na HR, et al. South Korean Study to Prevent Cognitive Impairment and Protect Brain Health Through Lifestyle Intervention in At-Risk Elderly People: Protocol of a Multicenter, Randomized Controlled Feasibility Trial. J Clin Neurol. 2020 Apr;16(2):292–303.
13. Sindi S, Thunborg C, Rosenberg A, Andersen P, Andrieu S, Broersen LM, et al. Multimodal Preventive Trial for Alzheimer’s Disease: MIND-ADmini Pilot Trial Study Design and Progress. J Prev Alzheimers Dis. 2022 Jan 1;9(1):30–9.
14. Tainta M, Ecay-Torres M, de Arriba M, Barandiaran M, Otaegui-Arrazola A, Iriondo A, et al. GOIZ ZAINDU study: a FINGER-like multidomain lifestyle intervention feasibility randomized trial to prevent dementia in Southern Europe. Alz Res Therapy. 2024 Feb 27;16(1):44.
15. Crivelli L, Calandri IL, Suemoto CK, Salinas RM, Velilla LM, Yassuda MS, et al. Latin American Initiative for Lifestyle Intervention to Prevent Cognitive Decline (LatAm-FINGERS): Study design and harmonization. Alzheimer’s & Dementia. 2023;19(9):4046–60.
16. Richard E, den Heuvel EV, Moll van Charante EP, Achthoven L, Vermeulen M, Bindels PJ, et al. Prevention of Dementia by Intensive Vascular Care (PreDIVA): A Cluster-randomized Trial in Progress. Alzheimer Disease & Associated Disorders. 2009 Sep;23(3):198.
17. Vellas B, Carrie I, Gillette-Guyonnet S, Touchon J, Dantoine T, Dartigues JF, et al. MAPT STUDY: A

MULTIDOMAIN APPROACH FOR PREVENTING ALZHEIMER’S DISEASE: DESIGN AND

BASELINE DATA. J Prev Alzheimers Dis. 2014 Jun;1(1):13–22.

18. Ponvel P, Shahar S, Singh DKA, Ludin AFM, Rajikan R, Rajab NF, et al. Multidomain Intervention for Reversal of Cognitive Frailty, Towards a Personalized Approach (AGELESS Trial): Study Design. Journal of Alzheimer’s Disease. 2021 Jul 21;82(2):673–87.
19. Liang CK, Lee WJ, Hwang AC, Lin CS, Chou MY, Peng LN, et al. Efficacy of Multidomain Intervention Against Physio-cognitive Decline Syndrome: A Cluster-randomized Trial. Archives of Gerontology and Geriatrics. 2021 Jul 1;95:104392.

**Items presented to experts for the content validity index**

| 1. | Does the module explain dementia vulnerability factors well? |
| --- | --- |
| 2. | Does the module adequately cover dementia prevention approach? |
| 3. | Do care bundle strategies effectively enhance elderly lifestyle? |
| 4. | Are all the diet information clear and specific? |
| 5. | Is the diet effectively emphasizing health benefits for diabetes and hypertension? |
| 6. | Is the language of the content clear, direct and specific? |
| 7. | According to you will these diet options achieve the goals they are meant for? |
| 8. | Are the suggested food groups in the diet plan correct and enough? |
| 9. | Are the pictures in the module clear, direct and specific? |
| 10. | Are the exercise descriptions clear, direct and specific? |
| 11. | Is the language of the content clear, direct and specific? |

| 12. | According to you will these exercises be doable among the elderly? |
| --- | --- |
| 13. | According to you will these exercises achieve the goals they are meant for? |
| 14. | Are the precautions suggested clear and enough? |
| 15. | Does the module sufficiently address substance use impacts on well-being? |
| 16. | Are de-addiction assessment methods accurate and clinically relevant? |
| 17. | Is substance use information clear and accurate in the module? |
| 18. | Do the cognitive reserve strategies need more clarity and specificity? |
| 19 | Do the module strategies align with current cognitive health research? |
| 20 | Is the module effectively emphasizing cognitive enhancement benefits for the elderly? |
| 21 | Does the module empower older adults for cognitive health choices? |
|  | Item 18 was reversed scored. |

**Disagreement of experts (Care Bundle Module Booklet)**

| Items with disagreement | Feedback | Action taken |
| --- | --- | --- |
| Are the suggested food groups in the diet plan correct and enough? | In page 34, it is written to Avoid sodium. Many elderly might avoid sodium  completely leading to complications of hyponatremia | We have mentioned appropriate salt quantity. |
| In your opinion, are these exercises doable among the elderly? | Some of the exercises / activities won't be possible in those with degenerative spine diseases. Each exercise should have a complete list of contraindications and  precautions; Many yoga postures are too complicated for elderly | We have added the list of contraindications and precautions |
| Are the precautions suggested clear and enough? | Some of the exercises / activities won't be possible in those with degenerative spine diseases. Each exercise should have a complete list of contraindications and  precautions | We have added the list of contraindications and precautions |
| According to you will these exercises achieve the goals they are meant for? | Many postures which are shown are just stretching postures which may not help |  |
| Do the cognitive reserve strategies need more clarity and specificity? | I don't think its appropriate to name some religious books as an examples, the recommendation can be more general | These examples have been modified. |

**Qualitative feedback by participants (CBM Booklet)**

| **Feedback** | **Changes Made** |
| --- | --- |
| **Rajasthan**   - Participants suggested simplifying language of plant protein sections. - For women, participants suggested adding demonstration by a female instead of male in physical exercise. - For alcohol consumption, instead of unit, quantity in ml or in any other form should be mentioned. | **Rajasthan**   - Language of plant protein section has been simplified in the booklet. - The concerns of the participants were heard, however experts in the project review committee advised to refrain from recording exercises of a female. - For alcohol consumption, edits were made in the booklet focusing on refraining from consumption of alcohol. |
| **Karnataka**   - Difficulty in comprehending certain words. - All the information and images in the SMRUTHI book are easily accessible to people making it a very useful guide. | **Karnataka**  Words were modified in the CBM booklet. |
| **Tripura**   - Pictures were not clear. - Could not understand some words. | **Tripura**   - Pictures were modified in content of Diabetes as suggested by participants. - Words were made simpler for the participants to understand. |
| **Himachal Pradesh**   - Could not understand some words in the booklet due to brighter colours in few pages. - Some pictures are difficult to understand. | **Himachal Pradesh**   - Colours in the respective pages have been modified so that the participants can read clearly. - Pictures in addiction section were made simpler for participants to understand better. |

**Qualitative feedback by participants (CBM Videos)**

| Feedback | Changes Made |
| --- | --- |
| Himachal Pradesh: Include more dietary items such as mustard leaves, maize flour bread. | Dietary items were added. (Video 6) |
| Himachal Pradesh: Engagement in cutting grasses, planting seeds, collecting water. | The feedback on ‘Physical Activity’ was added in the video. (Video 7) |
| Himachal Pradesh: Listening to religious, spiritual, folk music. Participating in collective religious practices. | The feedback on cognitive reserve was incorporated in the video. (Video 9) |
| Rajasthan: Include more dietary products in diet such as bajra, spinach, fenugreek. | Dietary products were added. (Video 6) |
| Rajasthan: Engagement in household activities, farming, walking, yoga, cycling, aerobic exercising. | The feedback on ‘Physical Activity’ was added in the video. (Video 7) |
| Rajasthan: Story-telling, playing games (e.g. sudoku), sorting clothes, reading newspapers, playing with grandchildren. | The feedback on cognitive reserve was incorporated in the video. (Video 9) |
| Tripura: Dental problems in some participants due to issues in chewing food such as nuts. Include vegetables such as bamboo shoots, rai saag, banana flowers (‘mocha’), fish, pumpkin leaf. | Alternative options to consume nuts, and suggested vegetables were added in the video. (Video 6) |
| Tripura: Engagement in brisk walking early morning at 4 am. | The feedback on ‘Physical Activity’ was added in the video. (Video 7) |
| Karnataka: Some participants were concerned about having dementia due to use of fertilizers in paddy crops. | Alternative option of separating dry and wet waste, and using wet waste as compost instead of using fertilizers was added in the video. (Diet Video 6) |
| Karnataka: Most individuals play indoor board games – Sudoku. | The feedback on cognitive reserve was incorporated in the video. (Video 9 Cognitive Reserve) |
| Karnataka: Engagement in walking and yoga. | The feedback on ‘Physical Activity’ was added in the video. (Video 7) |
| Karnataka: Both sunflower and mustard oil are expensive for people and most of them are able to buy and consume only palm oil. | Changes were made in the video to consume boiled vegetables or dishes instead of fried food. (Video 6) |
| Common feedback on Video 6 by most participants in all sites: Some of them got distracted when diet and diabetes were explained in Video 6 due to the length of the video. | Common feedback on Video 6 by most participants in all sites: For this, Video 6 has been divided into 2 parts: Video 6 part 1 explains the concept of diabetes and Video 6 part 2 explains diet. (Video 6) |
| Common Feedback | Participant verbatim (translated in English): Now what I think is and how to stay healthy, what foods we should use to reduce sugar, BP, from this project I got to know all the feelings and experiences.  The videos are interesting to watch. |

**Post-Intervention Adherence Scale (PIAS)**

**Post-Intervention Adherence Scale For De-Addiction**

1. Did you understand the importance of De-Addiction for preventing Dementia?

□	Yes	□	No

2. Did you get motivated to reduce substance use intake in your daily life?

□	Yes	□	No

3. Did you try to take steps to reduce substance use intake in your daily life?

□	Yes	□	No

4. Did you succeed or fail in your attempt to reduce substance use intake?

□ Succeed	□ Partially Succeed	□	Fail

5. What do you feel is the reason for your success or failure?

**Post-Intervention Adherence Scale For Cognitive Reserve**

1. Did you understand the importance of building Cognitive Reserve for preventing Dementia?

□	Yes	□	No

2. Did you get motivated to use techniques for building Cognitive reserve for preventing Dementia?

□	Yes	□	No

3. Did you try to take steps to build a Cognitive Reserve for preventing Dementia?

□	Yes	□	No

4. Which of the strategies were you able to perform during the period? Please tick (✔) one or more boxes.
   - Read a book
   - Narrated Stories
   - Play Board Games/ Card Games
   - Solving Puzzle together
   - Sort some items
   - Learn New skills
   - Tried cooking simple dishes
   - Listen to classical or Instrumental music
5. If yes, what are the number of days per week you performed techniques for building Cognitive Reserve?

□	1	□ 2	□ 3	□ 4	□ 5	□ 6	□ 7

6. For how many minutes a day, do you perform techniques to enhance Cognitive Reserve as suggested in the module?

□ More Than 30 Minutes	□ Less Than 30 Minutes	□ More Than 20 Minutes

□ Less Than 20 Minutes	□ Not at all

7. Did you succeed or fail in your attempt to perform Cognitive Reserve activities?

□ Succeed	□ Partially Succeed	□ Fail

8. Are you planning to continue building your Cognitive Reserve even after Intervention?

□	Yes	□ No

9. What do you feel is the reason for your success or failure?

**Post-Intervention Adherence Scale For Care Bundle Module Booklet**

1. Did you understand the content of the Care Bundle Module Booklet aimed for preventing Dementia?

□	Yes	□	No

2. Did you get motivated to use techniques mentioned in the Care Buncle Module Booklet?

□	Yes	□	No

3. Did you try to include the techniques mentioned in the Care Bundle Module Booklet in your daily routine?

□	Yes	□	No

4. Did you succeed or fail in your attempt to include techniques that are mentioned in the Care Bundle Booklet?

□ Succeed	□ Partially Succeed	□ Fail

5. What do you feel is the reason for your success or failure?
6. Did you understand the protective factors (Healthy Diet, Physical Activity, and Cognitive Reserve) of Dementia mentioned in the Care Bundle Module Booklet?

□	Yes	□ Partially	□ No

7. Did you understand the risk factors (Hypertension, Diabetes, Obesity, and Addiction) of Dementia mentioned in the Care Bundle Module Booklet?

□	Yes	□ Partially	□ No

8. Are you planning to continue techniques mentioned in the Care Bundle Module Booklet even after Intervention?

□Yes	□ No

9. Which of the Interventions were you able to perform during the period? Please tick (✔) one or more boxes.
   - Healthy Diet
   - Physical Activity
   - Cognitive Reserve
   - Deaddiction
   - Annual Screening

**Post-Intervention Adherence Scale For Physical Activities**

Please tick (✔) one box for each question.

1. Did you understand the importance of Physical Activities for preventing Dementia?

□ Yes	□ No

2. Did you get motivated to incorporate Physical Activities in your daily life?

□ Yes	□ No

3. Did you try to do the Physical Activities suggested in your daily life?

□ Yes	□ No

4. If yes, what are the number of days per week of physical activity?

□ 1	□ 2	□ 3	□ 4	□ 5	□ 6	□ 7

5. Which part of the Physical Activities module did you find easy to follow?

□ Physiotherapy	□ Yoga	□ Walking	□ Meditation	□ None

6. Which part of the Physical Activities module did you find difficult to follow?

□ Physiotherapy	□ Yoga	□ Walking	□ Meditation	□ None

5. For how many minutes do you walk daily as suggested in the module?

□ More Than 30 Minutes	□ Less Than 30 Minutes	□ More Than 20 Minutes

□ Less Than 20 Minutes	□ Not at all

6. Have you been following the exercise recommendations steps that we have suggested?

□ Always	□ Occasionally	□ Rarely	□ Very Rarely	□ Never

7. Have you been following the precautions that we have suggested?

□ Always	□ Occasionally	□ Rarely	□ Very Rarely	□ Never

8. Did you succeed or fail in your attempt in following the Physical Activity plan?

□ Succeed	□ Partially Succeed	□ Fail

9. What do you feel is the reason for your success or failure?
10. Do you see any positive changes in your physical health after following our Care Bundle Module?

□ Excellent	□ Very Good	□ Good	□ Fair	□ Poor

11. Do you feel you would continue these physical activities in future also?

□ Always	□ Occasionally	□ Rarely	□ Very Rarely	□ Never

12. Would you say that the care bundle module has positively influenced your physical	wellbeing through its suggested activities?
    - Yes
    - No
13. If yes, please describe._______________

| **Summary of content of the Care Bundle Module Booklet incorporated in the SMRUTHI-India Project.** | | | |
| --- | --- | --- | --- |
| **S.no.** | **Domain** | **Content** | **Sub-domains** |
| 1. | Psychoeducation | Dementia – its signs and symptoms | Pre-clinical phase  Mild Cognitive Impairment  10 signs of Dementia |
| 2. | Salient Modifiable factors | Diabetes  Obesity  Hypertension  Addiction | Detailed awareness about the adverse impact of these factors |
| 3. | Protective Factors | Diet Intervention | Components of a healthy diet  Customized diet charts (North, South, East, of India and Delhi) |
|  |  | Physical Exercise | Physiotherapy  Aerobic (Moderate and Vigorous)  Yoga |
|  |  | Cognitive Reserve | Activities to enhance mental well-being |
| 4. | Additional Information | Importance of Annual Screening process  Self-administered scales for assessing addiction |  |

| **Relevance items of ratings of items scale by five experts** | | | | | | | | | | | | |
| --- | --- | --- | --- | --- | --- | --- | --- | --- | --- | --- | --- | --- |
| **Item/ Response** | **E1**^[1]^ | **E2**^[2]^ | **E3**^[3]^ | **E4**^[4]^ | **E5** \|\| | **I-CVI** ¶ | **Total**  **Expert** | **Agree** | **UA**^#^ | **Pc**^[5]^ | **K**^[6]^ | **Interpretation** |
| Item 1 | 1 | 1 | 1 | 1 | 1 | 1 | 5 | 5 | 1 | 0·031 | 1 | Excellent |
| Item 2 | 1 | 1 | 1 | 1 | 1 | 1 | 5 | 5 | 1 | 0·031 | 1 | Excellent |
| Item 3 | 1 | 1 | 1 | 1 | 1 | 1 | 5 | 5 | 1 | 0·031 | 1 | Excellent |
| Item 4 | 1 | 1 | 1 | 1 | 1 | 1 | 5 | 5 | 1 | 0·031 | 1 | Excellent |
| Item 5 | 1 | 1 | 1 | 1 | 1 | 1 | 5 | 5 | 1 | 0·031 | 1 | Excellent |
| Item 6 | 1 | 1 | 1 | 1 | 1 | 1 | 5 | 5 | 1 | 0·031 | 1 | Excellent |
| Item 7 | 1 | 1 | 1 | 1 | 1 | 1 | 5 | 5 | 1 | 0·031 | 1 | Excellent |
| Item 8 | 1 | 1 | 1 | 1 | 0 | 0·8 | 5 | 4 | 0 | 0·156 | 0·763 | Excellent |
| Item 9 | 1 | 1 | 1 | 1 | 1 | 1 | 5 | 5 | 1 | 0·031 | 1 | Excellent |
| Item 10 | 1 | 1 | 1 | 1 | 1 | 1 | 5 | 5 | 1 | 0·031 | 1 | Excellent |
| Item 11 | 1 | 1 | 1 | 1 | 1 | 1 | 5 | 5 | 1 | 0·031 | 1 | Excellent |
| Item 12 | 0 | 1 | 1 | 1 | 0 | 0·6 | 5 | 3 | 0 | 0·312 | 0·418 | Fair |
| Item 13 | 1 | 1 | 1 | 1 | 0 | 0·8 | 5 | 4 | 0 | 0·156 | 0·763 | Excellent |
| Item 14 | 0 | 1 | 1 | 1 | 1 | 0·8 | 5 | 4 | 0 | 0·156 | 0·763 | Excellent |
| Item 15 | 1 | 1 | 1 | 1 | 1 | 1 | 5 | 5 | 1 | 0·031 | 1 | Excellent |
| Item 16 | 1 | 1 | 1 | 1 | 1 | 1 | 5 | 5 | 1 | 0·031 | 1 | Excellent |
| Item 17 | 1 | 1 | 1 | 1 | 1 | 1 | 5 | 5 | 1 | 0·031 | 1 | Excellent |
| Item 18 | 1 | 0 | 1 | 1 | 0 | 0·6 | 5 | 3 | 0 | 0·312 | 0·418 | Fair |
| Item 19 | 1 | 1 | 1 | 1 | 1 | 1 | 5 | 5 | 1 | 0·031 | 1 | Excellent |
| Item 20^[7]^ | 1 | 1 | 1 | 1 | 1 | 1 | 5 | 5 | 1 | 0·031 | 1 | Excellent |
| Item 21 | 1 | 1 | 1 | 1 | 1 | 1 | 5 | 5 | 1 | 0·031 | 1 | Excellent |
| Proportion Relevance | 0·904 | 0·952 | 1 | 1 | 0·81 |  |  |  |  |  |  |  |
| S-CVI^[8]^ / Average |  |  |  |  |  | **0·93** |  |  |  |  |  |  |
| Probability of Chance Occurrence (Pc) was computed using the formula: p_c_= [N! /A! (N -A)!] ^*^.5^N^where N= number of experts and A= number of panelists who agree that the item is relevant. Number of experts=5  Modified Kappa was computed using the formula: K= (I-CVI- P_C_)/(1- P_C_). Interpretation criteria for Kappa, using guidelines described in Cicchetti and Sparrow (1981): Fair=K of 0·40 to 0·59; Good=K of 0·60 to 0·74; and Excellent=K>0·74 | | | | | | | | | | | | |

| **Themes of CBM Videos** | | |
| --- | --- | --- |
| **S·N·** | **Video Theme** | **Description of Theme** |
| Video 1 | Introduction | Krishna is a 60+ year old man, residing in a rural setting. The introduction shows daily struggles of his forgetfulness (e.g. forgetting he has already fed the cattle), which is noted by his wife and son. |
| Video 2 | MCI^[9]^ and Dementia Overview | This video elaborates on the scene where Krishna’s son takes him to the doctor and the doctor explains the concepts of normal ageing, mild cognitive impairment and dementia. |
| Video 3 | Risk Factors | This video continues with the doctor’s scene when the son is curious about the causes of dementia and the doctor explains the risk factors (e.g. hypertension diabetes) associated with it. |
| Video 4 | Hypertension | This video elaborates on the concept of hypertension as explained by an Asha Worker who visits Krishna’s house and also teaches the son on how to measure blood pressure. It also highlights the importance of taking medication on time for blood pressure. |
| Video 5 | Risk Perception | This video highlights the lack of awareness around dementia in the rural settings. It includes the son explaining to fellow villagers about dementia, its risk factors, and its high prevalence in rural areas than urban areas. |
| Video 6 part I  Video 6 part II | Diabetes  Diet | This video is been divided into parts. The first part shows the doctor diagnosing Krishna with diabetes and explaining its cause and management. In the second part, the wife curiously asks the doctor about the diet that should be followed to prevent high sugar and cholesterol to which the doctor explains the food pyramid and diet schedule that should be followed. |
| Video 7 part I  Video 7 part II | Physical Activity | This video is divided in two parts. In the first part, Krishna and his wife meet a fellow villager (Bhoop Singh, a retired army officer) who is known in the village for his physical fitness. He explains the types of physical activity, emphasizing on stretching/strengthening exercises for different muscle groups and aerobic exercises. In the second part, we have recorded a live video demonstrating simple exercises which can be helpful in remaining physically active. |
| Video 8 | Addiction | This video includes a scene where Krishna is asked by his friends to try smoking hookah. Bhoop Singh happens to pass by and explains how addiction works and can be harmful in multiple ways including decline in cognitive health. |
| Video 9 | Cognitive Reserve | This video includes Krishna’s wife’s hesitation in engaging in something new like playing with her granddaughter. It continues with Asha Worker, highlighting the importance of being cognitively active and the concept of cognitive reserve in simple language. |
| Video 10 | Conclusion | This video highlights all the important points that have been discussed in previous videos, to give a summarized list of key takeaways.  Key takeaways have been added at the end of each video. |

**Proposed lifestyle intervention model for dementia prevention in at-risk elderly**

**Summary and agreement of responses from five experts**

 ^[10]^

16 items

Reached agreement

5 items

Did not reach agreement (supplementary appendix)

5 items

Amended based on feedback provided on open-ended comment box

**LIfestyle for BRAin health LIBRA INDEX**

**CONSORT 2025 checklist of information to include when reporting a randomised trial***

| **Section / Topic** | **No** | **CONSORT 2025 checklist item description** | **Section headings** | **First sentence** |
| --- | --- | --- | --- | --- |
| **Title and abstract** | | |  |  |
| Title and structured abstract | 1a | Identification as a randomised trial | Abstract | It was pilot tested for feasibility and efficacy in hospital setting using cohort multiple randomized controlled trial (cmRCT) design. |
|  | 1b | Structured summary of the trial design, methods, results, and conclusions | Abstract | It was pilot tested for feasibility and efficacy in hospital setting using cohort multiple randomized controlled trial (cmRCT) design.  Intervention was developed in three languages (Hindi, Kannada, Bengali) including risk and protective factors of dementia.  CBM demonstrated high content validity, acceptability, and adherence across hospital and rural settings. |
| **Open science** | | |  |  |
| Trial registration | 2 | Name of trial registry, identifying number (with URL) and date of registration | Abstract | The trial protocol was registered in Clinical Trials Registry – India (CTRI/2024/01/061172); first participant enrolled in pilot cmRCT on April 12, 2024 and in SMRUTHI Cohort on July 27, 2023. |
| Protocol and statistical analysis plan | 3 | Where the trial protocol and statistical analysis plan can be accessed | Introduction | The “Strategic Multimodal Intervention in at-risk Elderly Indians for Prevention of Dementia: SMRUTHI India” is an ongoing cohort study of the rural population (four zones of India) |
| Data sharing | 4 | Where and how the individual de-identified participant data (including data dictionary), statistical code and any other materials can be accessed | Data availability | The data is available on request from the corresponding author. |
| Funding and conflicts of interest | 5a | Sources of funding and other support (e.g., supply of drugs), and role of funders in the design, conduct, analysis and reporting of the trial | Funding and declaration of interest | The current study was funded by Indian Council of Medical Research (ICMR).  The current study was supported by Indian Council of Medical Research (ICMR), grant number [SWG/Neuro/51/TF Centre I/2021-NCD-I].  The authors declare no conflicts of interest. |
|  | 5b | Financial and other conflicts of interest of the manuscript authors | Declaration of interest | The authors declare no conflicts of interest. |
| **Introduction** | | |  |  |
| Background and rationale | 6 | Scientific background and rationale | Introduction | Dementia is a chronic neurodegenerative disease with significant health and socioeconomic concerns.  But there is paucity of studies targeting primary prevention of dementia in this region. |
| Objectives | 7 | Specific objectives related to benefits and harms | Introduction, methods | Based on the established cohort, the main aim of this study is to develop and validate a multimodal intervention for the primary prevention of dementia in the form of a booklet and videos (Care Bundle Module-CBM), evaluate feasibility and acceptability using qualitative data and quantitative tools [Patient Educational Material Assessment Tool (PEMAT)], and pilot test in the target population.  Any unintended harm during performing or consuming the suggested physical activities/diet were asked to be reported for the team to monitor. |
| **Methods** | | |  |  |
| Patient and public involvement | 8 | Details of patient or public involvement in the design, conduct and reporting of the trial | Methods | The study sample was derived from SMRUTHI cohort, whose recruitment was started on July 27, 2023. |
| Trial design | 9 | Description of trial design including type of trial (e.g., parallel group, crossover), allocation ratio, and framework (e.g., superiority, equivalence, non-inferiority, exploratory) | Methods | To assess the feasibility of the intervention, the pilot study conducted at the central site employed a methodology modelled on the original SMRUTHI cohort. This methodology follows a cohort multiple randomized controlled trial (cmRCT) design, in which participants are randomly assigned to the intervention arm. |
| Changes to trial protocol | 10 | Important changes to the trial after it commenced including any outcomes or analyses that were not prespecified, with reason | No changes | - |
| Trial setting | 11 | Settings (e.g., community, hospital) and locations (e.g., countries, sites) where the trial was conducted | Methods | It was tested in hospital and rural settings (Figure 1) |
| Eligibility criteria | 12a | Eligibility criteria for participants | Methods | The pilot study participants at central site included individuals aged ≥55 years, CAIDE score ≥6, and normal cognition (no complaints on Subjective Cognitive Decline – Interview and normal performance on Addenbrooke’s Cognitive Examination III/ACE III) at baseline (Figure 1) |
|  | 12b | If applicable, eligibility criteria for sites and for individuals delivering the interventions (e.g., surgeons, physiotherapists) | NA | - |
| Intervention and comparator | 13 | Intervention and comparator with sufficient details to allow replication. If relevant, where additional materials describing the intervention and comparator (e.g., intervention manual) can be accessed | Methods | The intervention arm was provided with the booklet and orientation was done by a team of field investigators and psychologists using an explanatory video.  The control arm received routine health advice in the form of a health awareness video. |
| Outcomes | 14 | Pre-specified primary and secondary outcomes, including the specific measurement variable (e.g., systolic blood pressure), analysis metric (e.g., change from baseline, final value, time to event), method of aggregation (e.g., median, proportion), and time point for each outcome | Methods | The primary outcome included change in the Lifestyle for Brain Health (LIBRA) Index at six months, and secondary outcomes included efficacy and feasibility of the intervention. |
| Harms | 15 | How harms were defined and assessed (e.g., systematically, non-systematically) | Methods | Any unintended harm during performing or consuming the suggested physical activities/diet were asked to be reported for the team to monitor. |
| Sample size | 16a | How sample size was determined, including all assumptions supporting the sample size calculation | Methods | The study sample was derived from SMRUTHI cohort, whose recruitment was started on July 27, 2023. |
|  | 16b | Explanation of any interim analyses and stopping guidelines | NA | - |
| Randomisation: |  |  |  |  |
| Sequence generation | 17a | Who generated the random allocation sequence and the method used | NA | - |
|  | 17b | Type of randomisation and details of any restriction (e.g., stratification, blocking and block size) | NA | - |
| Allocation concealment mechanism | 18 | Mechanism used to implement the random allocation sequence (e.g., central computer/telephone; sequentially numbered, opaque, sealed containers), describing any steps to conceal the sequence until interventions were assigned | NA | - |
| Implementation | 19 | Whether the personnel who enrolled and those who assigned participants to the interventions had access to the random allocation sequence | NA | - |
| Blinding | 20a | Who was blinded after assignment to interventions (e.g., participants, care providers, outcome assessors, data analysts) | Methods | The outcome assessors were blinded to the participants receiving the intervention or care as usual. |
|  | 20b | If blinded, how blinding was achieved and description of the similarity of interventions | NA | - |
| Statistical methods | 21a | Statistical methods used to compare groups for primary and secondary outcomes, including harms | Methods | In pilot feasibility study, to assess differences between pre- and post-intervention scores, change in measures were investigated using paired samples t-test (and summarised using mean ± SD) for normally distributed measures including MIND diet score, QOL, ACE III, and other ICMR-NCTB measures viz VLT (TL, Delayed Recognition), TNI-93 (Total & Spatial Recall), CF, PNT, score (for copy, immediate, and delayed) recall, and SDT score. |
|  | 21b | Definition of who is included in each analysis (e.g., all randomised participants), and in which group | Methods, Result Tables | Moreover, analyses were performed using complete-case data; participants who did not complete the follow-up assessment were excluded from the outcome analyses (Table 2 and Table 3). |
|  | 21c | How missing data were handled in the analysis | Methods | Moreover, analyses were performed using complete-case data; participants who did not complete the follow-up assessment were excluded from the outcome analyses. |
|  | 21d | Methods for any additional analyses (e.g., subgroup and sensitivity analyses), distinguishing prespecified from post-hoc | NA | - |
| **Results** | | |  |  |
| Participant flow, including flow diagram | 22a | For each group, the numbers of participants who were randomly assigned, received intended intervention, and were analysed for the primary outcome | Results | A total of 33 participants were enrolled at central site and 20 at periphery sites.  At the central site, in intervention group (n=8), LIBRA index improved significantly from −3·0 (−4·6,0·2) to −4·6 (−5·4, −4·55; p=0·02) (Table 3). |
|  | 22b | For each group, losses and exclusions after randomisation, together with reasons | Results | However, retention rate was lower (42·4%), attributable to lack of feasibility of participants traveling to central site. |
| Recruitment | 23a | Dates defining the periods of recruitment and follow-up for outcomes of benefits and harms | Methods | Participant enrolment for the pilot cmRCT commenced on April 12, 2024. |
|  | 23b | If relevant, why the trial ended or was stopped | NA | - |
| Intervention and comparator delivery | 24a | Intervention and comparator as they were actually administered (e.g., where appropriate, who delivered the intervention/comparator, how participants adhered, whether they were delivered as intended [fidelity]) | Results | The intervention arm was provided with the booklet and orientation was done by a team of field investigators and psychologists using an explanatory video.  The control arm received routine health advice in the form of a health awareness video.  Adherence of eight participants was assessed: ≥2 of the five intervention components was high, with full participation in cognitive activities (100%), physical activity (75%), and dietary changes (75%).  Adherence to ≥2 intervention components remained high, with 100% engagement in cognitive activities, 88·2% in physical activity, and 70·6% in dietary changes. |
|  | 24b | Concomitant care received during the trial for each group | NA | - |
| Baseline data | 25 | A table showing baseline demographic and clinical characteristics for each group | Result table | Table 1: Mean ± SD / Median [IQR] for Sociodemographic characteristics of participants |
| Numbers analysed,  outcomes and estimation | 26 | For each primary and secondary outcome, by group:  - the number of participants included in the analysis  - the number of participants with available data at the outcome time point  - result for each group, and the estimated effect size and its precision (such as 95% confidence interval)  - for binary outcomes, presentation of both absolute and relative effect size | Results | At the central site, in intervention group (n=8), LIBRA index improved significantly from −3·0 (−4·6,0·2) to −4·6 (−5·4, −4·55; p=0·02) (Table 3).  At central site, recruitment rate was 12·5% of pre-screened individuals.  The median LIBRA index (n=17) improved from 0·55 (−0·8, 1·7) to −1·0 (−1·4, 0·6; p=0·001) at Periphery (Table 3).  Adherence of eight participants was assessed: ≥2 of the five intervention components was high, with full participation in cognitive activities (100%), physical activity (75%), and dietary changes (75%).  At periphery sites, recruitment rate was higher with a six-month retention rate of 85% (3/20 dropouts). |
| Harms | 27 | All harms or unintended events in each group | Results | No harm was reported by any participant during the intervention. |
| Ancillary analyses | 28 | Any other analyses performed, including subgroup and sensitivity analyses, distinguishing pre-specified from post-hoc | NA | - |
| **Discussion** | | |  |  |
| Interpretation | 29 | Interpretation consistent with results, balancing benefits and harms, and considering other relevant evidence | Discussion | These findings are consistent with prior multidomain intervention trials (2), which indicate that programs addressing physical activity, diet, cognitive engagement, and vascular risk control can produce short-term reductions in risk factors and potentially delay cognitive decline. |
| Limitations | 30 | Trial limitations, addressing sources of potential bias, imprecision, generalisability, and, if relevant, multiplicity of analyses | Discussion | The major limitation of this approach is heavy reliance on psychoeducation and whether a less intensive approach may successfully achieve sustained behaviour modification. |

*We strongly recommend reading this statement in conjunction with the CONSORT 2025 Explanation and Elaboration and/or the CONSORT 2025 Expanded Checklist for important clarifications on all the items. We also recommend reading relevant CONSORT extensions. See [www.consort-spirit.org](http://www.consort-spirit.org).

Citation: Hopewell S, Chan AW, Collins GS, Hróbjartsson A, Moher D, Schulz KF, et al. CONSORT 2025 Statement: updated guideline for reporting randomised trials. BMJ. 2025; 388:e081123. <https://dx.doi.org/10.1136/bmj-2024-081123>.

© 2025 Hopewell et al. This is an Open Access article distributed under the terms of the Creative Commons Attribution License (<https://creativecommons.org/licenses/by/4.0/>), which permits unrestricted use, distribution, and reproduction in any medium, provided the original work is properly cited.

1. Expert 1
2. Expert 2
3. Expert 3
4. Expert 4

   || Expert 5

   ¶ Item Level Content Validity Index

   # Universal Agreement
5. Probability of chance occurrence
6. Modified Kappa
7. Reversed items on the scale
8. Scale wise Content Validity Index
9. MCI- Mild Cognitive Impairment
10. Item 20 was reverse scored
